# CO Rebinding Kinetics and Molecular Dynamics Simulations Highlight Dynamic Regulation of Internal Cavities in Human Cytoglobin

**DOI:** 10.1371/journal.pone.0049770

**Published:** 2013-01-04

**Authors:** Matteo Gabba, Stefania Abbruzzetti, Francesca Spyrakis, Flavio Forti, Stefano Bruno, Andrea Mozzarelli, F. Javier Luque, Cristiano Viappiani, Pietro Cozzini, Marco Nardini, Francesca Germani, Martino Bolognesi, Luc Moens, Sylvia Dewilde

**Affiliations:** 1 Institute of Complex Systems - Molekulare Biophysik (ICS-5) Forschungszentrum Jülich, Jülich, Germany; 2 Dipartimento di Fisica e Scienze della Terra, Università degli Studi di Parma, Parma, Italy; 3 Dipartimento di Scienze degli Alimenti, Università degli Studi di Parma, Parma, Italy; 4 INBB, Biostructures and Biosystems National Institute, Rome, Italy; 5 Departament de Fisicoquímica and Institut de Biomedicina (IBUB), Facultat de Farmàcia, Universitat de Barcelona, Barcelona, Spain; 6 Dipartimento di Farmacia, Università degli Studi di Parma, Parma, Italy; 7 NEST, Istituto Nanoscienze-CNR, Pisa, Italy; 8 Dipartimento di BioScienze, CNR-IBF, and CIMAINA, Università degli Studi di Milano, Milano, Italy; 9 Department of Biomedical Sciences, University of Antwerp, Antwerp, Belgium; University of South Florida College of Medicine, United States of America

## Abstract

Cytoglobin (Cygb) was recently discovered in the human genome and localized in different tissues. It was suggested to play tissue-specific protective roles, spanning from scavenging of reactive oxygen species in neurons to supplying oxygen to enzymes in fibroblasts. To shed light on the functioning of such versatile machinery, we have studied the processes supporting transport of gaseous heme ligands in Cygb. Carbon monoxide rebinding shows a complex kinetic pattern with several distinct reaction intermediates, reflecting rebinding from temporary docking sites, second order recombination, and formation (and dissociation) of a bis-histidyl heme hexacoordinated reaction intermediate. Ligand exit to the solvent occurs through distinct pathways, some of which exploit temporary docking sites. The remarkable change in energetic barriers, linked to heme bis-histidyl hexacoordination by HisE7, may be responsible for active regulation of the flux of reactants and products to and from the reaction site on the distal side of the heme. A substantial change in both protein dynamics and inner cavities is observed upon transition from the CO-liganded to the pentacoordinated and bis-histidyl hexacoordinated species, which could be exploited as a signalling state. These findings are consistent with the expected versatility of the molecular activity of this protein.

## Introduction

Shortly after the discovery of neuroglobin (Ngb), a globin mainly expressed in vertebrate nervous tissues and the retina [1], a fourth vertebrate globin was isolated in rat stellate cells using a proteomic approach, and was named STAP (stellate cell activation-associated protein) [2]. This novel heme protein exhibited peroxidase activity toward hydrogen peroxide and linoleic acid hydroperoxide. Almost independently, the sequence of this globin was identified in mouse, man and zebrafish and, given that it is expressed in all types of human tissues, was eventually termed cytoglobin (Cygb) [3]. Human Cygb consists of 190 amino acids, showing extensions of about 20 amino acids at both C- and N-termini with respect to standard globins. The amino acid sequence fits well into the conserved globin fold pattern, covering helices from A to H, and key residues such as the proximal (F8) and distal (E7) histidines and phenylalanine CD1, at the CD corner [4]. Interestingly, Cygb shares about 30% amino acid sequence identity with myoglobin (Mb), suggesting that Cygb and Mb diverged from a common ancestor [3].

Together with Ngb, Cygb is the first example of bis-histidyl hexacoordinated globin in humans and other vertebrates. In the absence of exogenous ligands, the sixth heme iron coordination position is occupied by the distal HisE7 residue in both the ferric and ferrous forms [5], [6]. Bis-histidyl hexacoordinated hemoglobins (Hbs) have been found in animals, cyanobacteria [7] and plants [8], and in their deoxy Fe^2+^ state they reversibly bind exogenous diatomic ligands, usually with high affinities [9], [10]. Binding of exogenous ligands is possible only with concomitant displacement of the distal HisE7, a regulatory mechanism which has been suggested to require a substantial conformational change [11], [12]. Despite the widespread occurrence of bis-histidyl hexacoordinated Hbs, their physiological role is as yet largely unknown, although recent studies have suggested their involvement in NO detoxification by acting as NO scavengers, playing a protective role during hypoxia [8], [13], [14], [15].

Cygb displays some peculiar structural features, like the N- and C-terminal extensions, which might mediate specific protein–protein interactions [4]. In addition, the crystal structure of bis-histidyl hexacoordinated Cygb shows an extended apolar protein matrix cavity, connected to the exterior through a narrow tunnel nestled between helices G and H. On the other hand, the internal cavity differs from those recognized to play a functional role in Mb, Ngb, truncated Hb and *C. lacteus* mini-Hb [16], and may provide a special “ligand tunnelling” pathway [4] that raises intriguing questions about the functional role of Cygb. Understanding the involvement of cavities and conformational changes in modulating ligand binding is crucial for the comprehension of the mechanisms that are relevant to the function(s) of Hbs. Although several hypotheses have been proposed for Cygb, so far no clear-cut evidence has been obtained in favour of any of them. Besides a role in NO or reactive oxygen species scavenging, [4], [17], [18] Cygb has been suggested to act as an oxygen storage or sensor protein [19], or as an enzyme involved in collagen synthesis [4]. A role in cancer as a tumor suppressor gene has also been discussed, following the finding that the promoter region of the gene encoding for Cygb is hypermethylated and as such underexpressed in tumours [20].

In order to understand the accessibility of internal cavities to small gaseous ligands and explain how this accessibility is tuned by HisE7 hexacoordination, we have performed flash photolysis experiments on CO complexes of Cygb in solution and gels [21], [22]. Thermodynamically unfavoured protein conformations have been selectively stabilized through silica gel encapsulation [23], [24], [25], and functional properties of structurally distinct conformations associated with CO-liganded, pentacoordinated, and bis-histidyl hexacoordinated structures have been determined [26]. Analyses of molecular dynamics simulations, together with the crystal structure of the HisE7Gln mutant, have allowed us to study the shape and connectivity of internal cavities. Our results highlight structural and functional features capable of providing versatility to this molecular machine.

## Materials and Methods

### Sample preparation

Wild type (wt) human Cygb and the His81(E7)Gln mutant were heterologously expressed as previously described [27] (the His81(E7)Gln mutant bears mutations Cys38(B2)Ser and Cys83(E9)Ser for crystallization purposes and will be denoted hereafter HE7Q Cygb*). Laser flash photolysis experiments on the wt protein were performed on reduced samples (where the Cys38-Cys83 disulfide bond is broken), prepared by reacting the purified proteins with 10 mM dithiothreitol (DTT) overnight. The mutant HE7Q Cygb* was not subject to DTT treatment, as mutations Cys38Ser and Cys83Ser prevent formation of any intramolecular disulfide bridge. Encapsulation of Cygb in silica gels was carried out following a previously described protocol (see Experimental Methods in Supporting Information S1) [28].

### Kinetic experiments and data analysis

The CO rebinding curves were measured by monitoring changes in absorbance at 436 nm or by measuring transient spectra in the Soret band after nanosecond laser photolysis at 532 nm with a previously described setup [23]. Repetition rate of laser pulses was kept at 0.1 Hz for the wt Cygb, and 2 Hz for the HE7Q Cygb* mutant. Lifetime distributions associated with ligand rebinding kinetics were determined with a maximum entropy method (MEM; see Experimental Methods in Supporting Information S1) [29]. Differential equations associated with the kinetic ligand migration mechanism were solved and optimized numerically [24] (see Supporting Information S1 for fitting details). In order to improve the retrieval of microscopic rate constants, data from flash photolysis at two different CO concentrations and at the same temperature were simultaneously fitted. This global analysis was repeated at several different temperatures between 10°C and 45°C. The activation parameters for the microscopic rate constants were determined from the resulting linear Eyring plots (Tables S1, S2, S3, S4 in Supporting Information).

### X-ray structural analysis of HE7Q Cygb*

Crystals of HE7Q Cygb* mutant were grown using the hanging drop vapour diffusion setup (see Experimental Methods in Supporting Information S1; data processing statistics are reported in Table S5). The crystals diffracted up to 2.8 Å resolution using synchrotron radiation (beam line ID23-2, ESRF, Grenoble, France), and were shown to belong to the orthorhombic space group *P*2_1_2_1_2_1_, with unit cell parameters *a* = 48.8 Å, *b* = 70.1 Å, *c* = 102.1 Å, α = β = γ = 90.0° (two protein molecules in the asymmetric unit). The HE7Q Cygb* structure was determined by molecular replacement using the program Phaser [30] (see see Experimental Methods in Supporting Information S1 for details). In the end of the refinement stages, 20 water molecules, 2 ferricyanide molecules, 2 cyanide and 1 acetate ions were located. For the two molecules in the asymmetric unit, no interpretable electron density was present for the N-terminal (residues 1–17) and C-terminal (residues 172–190) regions. The final R-factor and R-free values were 20.7% and 27.7%, respectively.

### MD simulations

MD simulations were run for human Cygb in bis-histidyl hexacoordinated (*Cygb_h_*), pentacoordinated (*Cygb_p_*) and oxygenated (O_2_Cygb) states using the parmm99SB force field [31] and the Amber9 package [32]. *Cygb_h_* was modelled using as template the X-ray structure 1UT0 (solved at 2.40 Å) [33]. Two templates were used to build up the simulation systems for both *Cygb_p_* and O_2_Cygb. The first template was the X-ray structure of the HE7Q Cygb* mutant, which contains cyanide in the distal cavity above the heme (see below for details). For our purposes here, the mutated residues Gln(E7)81, Ser38 and Ser83 were restored to the native amino acids (His81, Cys38 and Cys83). The second template was the X-ray structure 3AG0 (solved at 2.60 Å), which contains CO bound to the heme [34]. In the two cases the ligand (cyanide, CO) was removed to simulate *Cygb_p_*, or replaced by O_2_ to simulate O_2_Cygb. Thus, four distinct trajectories were sampled for pentacoordinated and oxygenated states: *Cygb_p_*(HE7Q), *Cygb_p_*(3AG0), O_2_Cygb(HE7Q) and O_2_Cygb(3AG0).

The overall fold of the three X-ray structures (1UT0, 3AG0 and the HE7Q Cygb* mutant) is very similar, as noted in a root-mean square deviation (rmsd) of the backbone C_α_ atoms in the range 0.5–0.7 Å. However, there are two main differences. As expected, the presence of the ligand in the distal cavity changes the conformation of HisE7, as the torsion N-Cα-Cβ-C4 varies from −175.2 degrees in 1UT0 to −53.9 degrees in 3AG0. A more intriguing difference concerns the conformation of Trp151, which is found in two conformations (see Figure S1 in Supporting Information). In both 1UT0 and the HE7Q Cygb* mutant, the torsional angles N-Cα-Cβ-C3 and Cα-Cβ-C3-C3α are about −165 and 85 degrees. However, the alternative conformation found in 3AG0 is characterized by torsion angles of −88 and −82 degrees, respectively. Therefore, the distinct simulation models allows us to explore the effect of the conformational variability of Trp151. On the other hand, although Cygb is generally found as a crystallographic dimer, the extension of the contact surface (760 Å^2^) [33] appears insufficient to provide a stabilization of the dimer in solution. In fact, recent experimental data demonstrate that the protein is monomeric in dilute solutions, with extended N- and C-terminal regions [35]. Therefore, in all cases, MD simulations were run on monomeric proteins.

Each system was simulated for 100 ns trajectories, collecting frames at 1 ps intervals. Details of the MD simulations, including preparation of simulated systems, thermalization and simulation protocol, are given in Supporting Information S1 (see Experimental Methods).

Essential dynamics was used to analyze the dynamical behavior of the protein backbone [36], [37]. MDpocket [38] was used to detect cavities in the protein matrix. Calculations were performed on 10^3^ snapshots taken regularly in the regions 20–60 ns and 60–100 ns of the trajectory. High-density isocontours show stable cavities found during the trajectory, while low-density values reflect transient or nearly non-existent pockets. The similar distribution of cavities found for the two time-windows support the structural integrity of the trajectory. Finally, Implicit Ligand Sampling (ILS) [39] was used as an alternative approach to identify inner cavities favorable for ligand docking and migration. ILS computations were performed using a 3D grid that encompasses the whole protein with a 0.5 Å resolution. Moreover, 50 orientations of the probe oxygen molecule per grid point and a total set of 10^4^ snapshots taken every 4 ps using the same time-windows considered for MDpocket analysis.

### NO dioxygenase activity

The rate of NO dioxygenase activity was determined by rapid mixing using a stopped-flow apparatus (SX.18MV, Applied Photophysics). A solution containing 100 mM phosphate, 6 µM O_2_Cygb at pH 7.0 was prepared by addition of sodium ascorbate at a concentration of 10 mM in presence of 5 UI/ml catalase under strictly anaerobic conditions. Upon completion of the reduction, the protein solution was quickly exposed to a 100% oxygen atmosphere and immediately loaded on the stopped-flow apparatus. A stock solution containing ∼1 mM NO was generated by anaerobically dissolving MAHMA NONOate in a deoxygenated 100 mM phosphate solution at pH 7.0. A ∼20 µM solution of NO was obtained by dilution under anaerobic conditions. The exact concentration of NO was then measured by titration with deoxygenated human haemoglobin A (HbA) and determined to be 18 µM. The 6 µM O_2_Cygb and 18 µM NO solutions were mixed and the reaction was monitored at 419 nm. 5 traces were collected and averaged. All measurements were carried out at 20°C. For comparison, the same NO solution was reacted with a 6 µM O_2_HbA solution. The instrument dead time is about 1 ms.

## Results

### CO rebinding kinetics to Cygb in solution and in silica gels

**Figure 1:**
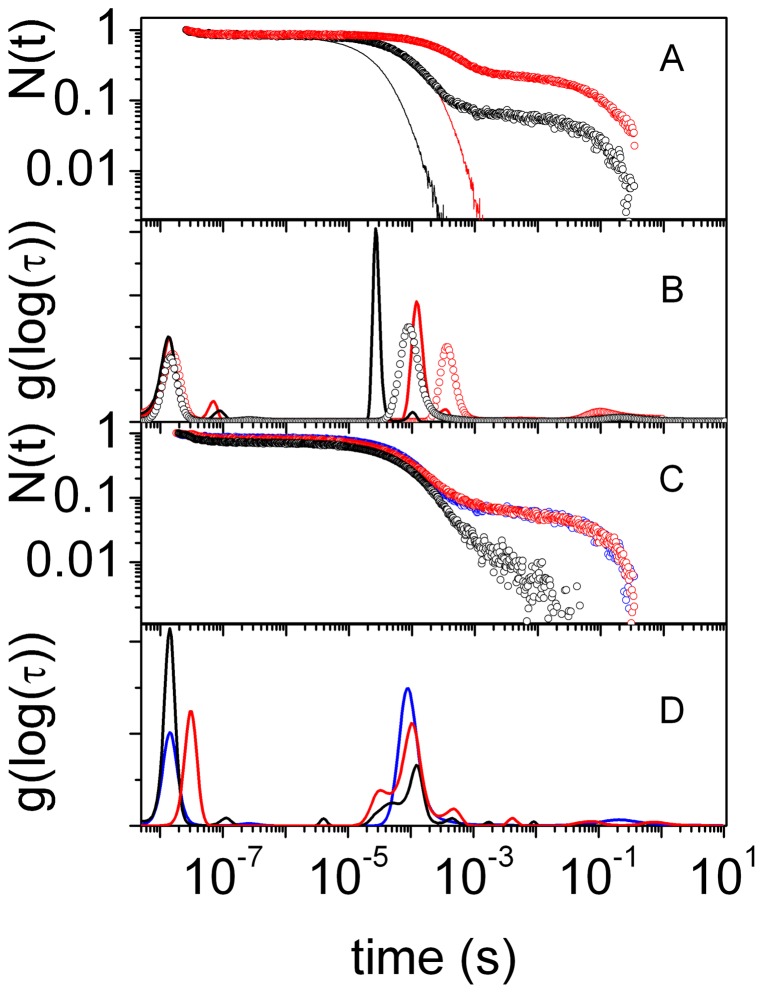
CO rebinding kinetics. (A) Comparison between the CO rebinding kinetics to wt Cygb (circles) and HE7Q Cygb* (solid lines) solutions at 40°C, equilibrated with 1 atm CO (black) and 0.1 atm CO (red). (B) Lifetime distributions associated with the rebinding kinetics in panel A. (C) Comparison between the CO rebinding kinetics to wt Cygb solutions (blue circles), wt COCygb gels (black circles) and wt Cygb+CO gels (red circles). T = 40°C, 1 atm CO. (D) Lifetime distributions associated with the rebinding kinetics in panel C.

The CO rebinding kinetics to Cygb solutions (Figure 1A) reveals a complex kinetic pattern in which three phases can be distinguished. After photolysis, substantial geminate rebinding is observed on the nanosecond time scale, followed by a biphasic bimolecular phase. The faster process in the bimolecular phase is associated with rebinding to pentacoordinated Cygb molecules (*Cygb_p_*), while the slower one is due to rebinding to Cygb molecules that have switched to the bis-histidyl hexacoordinated species (*Cygb_h_*). Decay of the latter reaction intermediate is much slower since the apparent rate is determined by the distal HisE7 dissociation rate.

The above outlined kinetic features are shared with human Ngb [21], [40], although there are differences in the relative extent of the different phases. In particular, the geminate phase is much larger for Cygb than for Ngb, suggesting a higher reactivity and/or hindered escape to the solvent for the former. Inspection of the kinetic phases detected by the MEM analysis (Figure 1B) reveals the existence of multiple kinetic steps, several of which are essentially CO concentration-independent. A clear-cut thermal activation is also recognizable in many of these steps (see Figure S7 in Supporting Information).

Proper identification of reaction intermediates is fundamental to quantitatively describe the kinetics of ligand binding [24]. The bimolecular rebinding to *Cygb_p_* is easily identified thanks to the CO concentration dependence of this step. For example, in Figure 1B the band peaked at 90 ms (at 40°C and 1 atm CO; black curve) shifts to 350 ms when CO concentration is reduced tenfold (grey curve). The identity of the long-lived reaction intermediate can be demonstrated by mutating the distal His to a different amino acid, unable to coordinate to the heme Fe. In fact, the CO rebinding kinetics to the HE7Q Cygb* mutant completely lacks the slowest phase (Figure 1A and 1B), thus confirming the bis-histidyl identity of this reaction intermediate. The progress curves in Figure 1A also highlight a much faster second order rebinding for HE7Q Cygb* than for the wt protein.

In order to expose the different reactivities of penta- and bis-histidyl hexacoordinated structures, we took advantage of the silica gel encapsulation methodology to stabilize those two conformations, and determined the corresponding CO rebinding kinetics [41]. Following a well established protocol [28], [42], [43], Cygb was encapsulated either as the carbonmonoxy adduct (COCygb gels) or as the deoxy form (Cygb gels) to trap the liganded and unliganded structures, respectively. The latter were exposed to CO immediately before performing the laser flash photolysis experiments (Cygb+CO gels). Given the capability of silica gels to trap the three dimensional features of liganded and unliganded structures, the resulting rebinding kinetics after laser photolysis expose the different functional properties of the two molecular species [44]. As shown in Figure 1C, rebinding kinetics observed for COCygb and Cygb+CO gels are dramatically different. Visual inspection of the rebinding curves shows that when Cygb is encapsulated as COCygb, the physical constraints imposed by the gel highly inhibit bis-histidyl hexacoordination by the distal His. The gel also inhibits escape of the photodissociated ligand to the solvent phase, increases geminate recombination, and favours migration to internal docking sites. On the other hand, the rebinding kinetics to Cygb+CO gels is almost identical to the one observed in solution, indicating that formation of *Cygb_h_* is not hindered in the gel.

**Figure 2:**
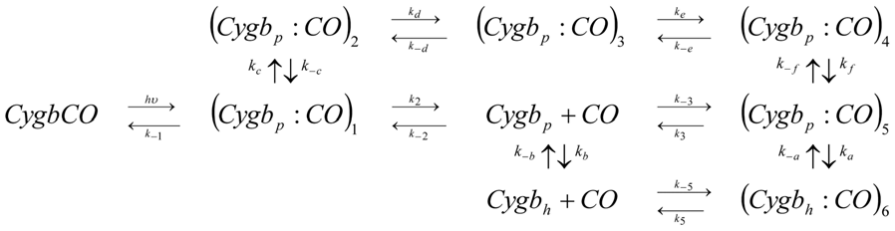
Minimal reaction scheme for the observed kinetics. After photodissociation of the CO complex of Cygb (*CygbCO*), the ligand can migrate to a primary docking site (*Cygb_p_:CO*)_1_, from which it can sequentially access secondary sites (*Cygb_p_:CO*)_i_, i = 2,…,5, or exit to the solvent (*Cygb_p_*). The deoxy, pentacoordinated species (*Cygb_p_*) is in equilibrium with the deoxy, bis-histidyl hexacoordinated species (*Cygb_h_*). Migration to the last reaction intermediate (*Cygb_h_:CO*)_6_ is concurrent to formation of the bis-histidyl complex.

**Figure 3:**
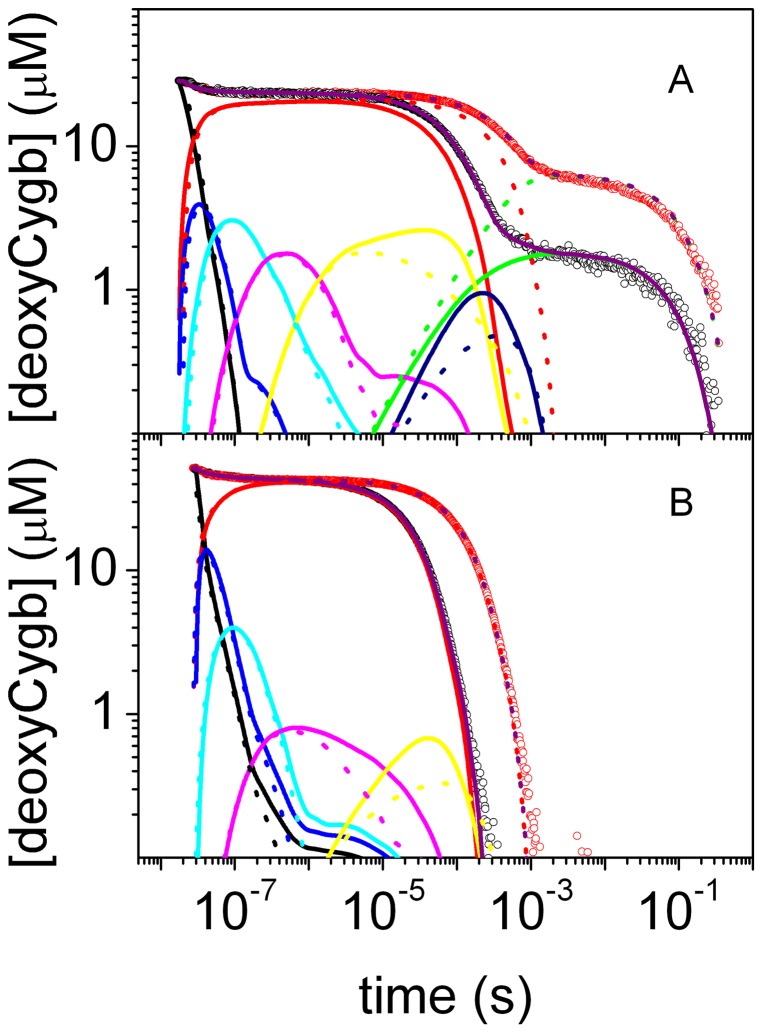
Kinetic analysis of CO rebinding. Analysis of the CO rebinding kinetics to wt Cygb (A) and HE7Q Cygb* mutant (B) solutions equilibrated with 1 atm CO (black circles) and 0.1 atm CO (red circles). T = 40°C. The fits (purple lines) are superimposed to the experimental data (circles). In the figures we have also reported the time course of the other relevant species in the scheme in Figure 2 at 1 atm CO (solid lines) and 0.1 atm (dotted lines): (*Cygb_p_:CO*)_1_ (black), (*Cygb_p_:CO*)_2_ (blue), (*Cygb_p_:CO*)_3_ (cyan), (*Cygb_p_:CO*)_4_ (magenta), (*Cygb_p_:CO*)_5_ (yellow), (*Cygb_h_:CO*)_6_ (dark blue), *Cygb_h_* (green), *Cygb_p_* (red).

The simplest reaction path that proved consistent with the observed kinetics under the tested conditions (at different CO concentrations, temperatures, in solution and encapsulated in silica gels) is outlined in Figure 2. Figure 3 shows the results of the fits with the kinetic model reported in Figure 2 to selected rebinding curves for wt Cygb and HE7Q Cygb* solutions (the microscopic rate constants at 20°C and the corresponding activation energies retrieved from Eyring plots are reported in Tables S1, S2, S3, S4 in Supporting Information). The time course can be perfectly reproduced under all investigated conditions, including the cases of the HE7Q Cygb* mutant as well as COCygb and Cygb+CO gels (see Figures S8, S9, S10, S11 for representative analyses).

Formation and decay of *Cygb_h_* was found to occur with microscopic rates *k*_b_ = 149 s^−1^ and *k*_−b_ = 1.8 s^−1^ at 20°C, which are slightly lower than the values reported by Trent et al. (*k*_b_ = 430 s^−1^ and *k*_−b_ = 5 s^−1^) [5], although obtained with a different method. The resulting equilibrium binding constant is 83, in agreement with the literature value of 86 [5]. In spite of the fact that the rate *k*_b_ is much lower than the value reported for human Ngb (*k*_b_ = 2000 s^−1^) [5], bis-histidyl hexacoordination occurs to a larger extent for Cygb than for Ngb after photolysis of their CO complexes, due to the very different values of the CO rebinding rates. CO rebinding to *Cygb_p_* from the solvent occurs with rate *k*_−2_ = 3.04×10^7^ M^−1^ s^−1^, in comparison with the value *k*_−2_ = 7.1×10^8^ M^−1^ s^−1^ observed for human Ngb [21]. Using the determined microscopic rate constants, the on-rate kinetic constant (*k*_ON_) is estimated to be 6.3×10^6^ M^−1^ s^−1^, which is in line with the literature value of 5.6×10^6^ M^−1^ s^−1^ [5] and is smaller than the values observed for human [21], [45], [46] and murine [46], [47], [48] Ngb. The slower reaction of CO with *Cygb_p_* thus favors bis-histidyl hexacoordination by the distal His, which occurs in higher yield than for Ngb. The gel has an appreciable effect on the value of *k*_ON_ with a twofold increase for COCygb gels when compared to Cygb solutions. As expected, no change at all is observed for Cygb+CO gels. A remarkable enhancement in *k*_ON_ is observed for the HE7Q Cygb* mutant, for which the estimated value is 2.96×10^7^ M^−1^ s^−1^.

Interestingly, the innermost rebinding step occurs with the same rate in Cygb and Ngb, *k*_−1_ = 1.5×10^7^ M^−1^ s^−1^, while the exit rates to the solvent and the migration rates to the first docking site are different (for Cygb *k*_2_ = 4.2×10^7^ s^−1^, *k*_c_ = 1.5×10^7^ s^−1^; for Ngb *k*_2_ = 1.4×10^8^ s^−1^, *k*_c_ = 5.5×10^7^ s^−1^). This has straightforward consequences on the geminate rebinding, which is larger for Cygb, mostly due to a lower escape probability from the primary docking site. Finally, while the rate *k*_−1_ undergoes only a minor increase when Cygb is trapped in COCygb gels, the exit rate *k*_2_ drops to 2.1×10^7^ s^−1^ (twofold decrease).

### X-ray structure of the HE7Q Cygb* mutant

The two subunits (A, B) found in the X-ray structure are assembled in a dimer identical to that found for native Cygb* [33]. Superimposition of the C_α_ atoms in the protein core of the two subunits reveals a strong structural conservation (rmsd of 0.45 Å), with limited differences in the G-H loop (residues 137–146). The cyanide ligand in the distal site pocket is found almost parallel to the heme plane (distance of 3.22 and 2.87 Å found in subunits A and B of the X-ray structure; Figure S2), oriented roughly along the line connecting the pyrrole N_A_ and N_C_ nitrogen atoms. This arrangement indicates that the structure better fits a pentacoordinated protein. The absence of ligand coordination likely arises from X-ray-induced Fe(III)→Fe(II) reduction, resulting essentially in loss of heme affinity for the ligand [49], as has been noticed for other heme proteins [50].

**Figure 4:**
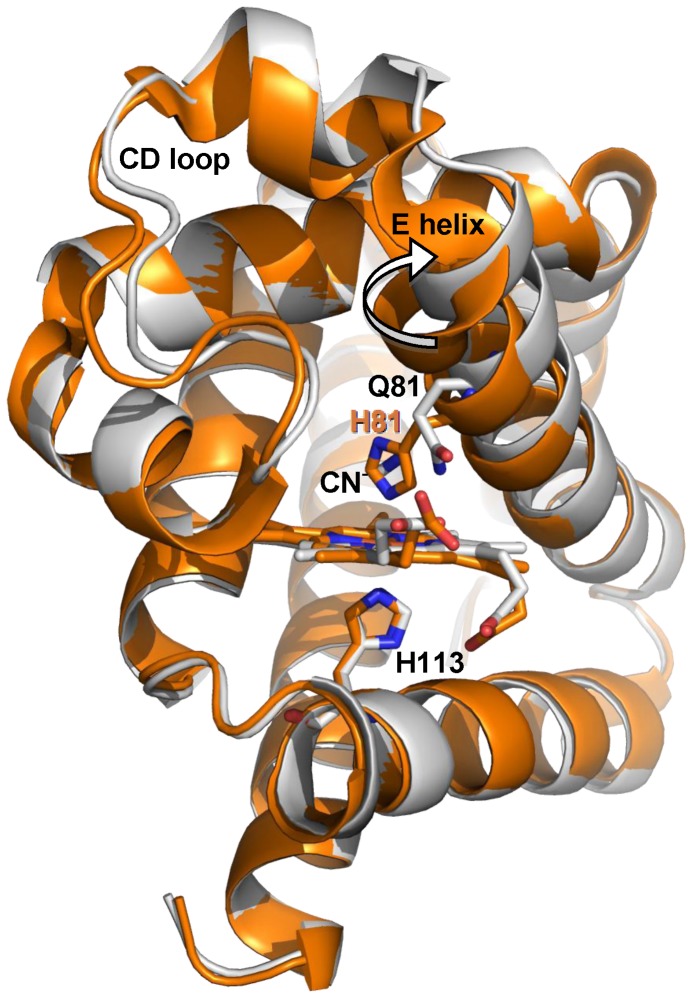
Superimposition of X-ray structures 1UT0 and HE7Q mutant. Representation of the backbone of HE7Q Cygb* mutant (gray ribbon) and Cygb* in the endogenous bis-histidyl hexacoordinated state (subunit A, orange ribbon). The rigid body movement of helix E is indicated by an arrow. The HE7Q Cygb* heme group is in red colour. For clarity, the CN^−^ ion (not coordinated to the heme iron) is omitted from the HE7Q Cygb* distal cavity (see Figure S2). Relevant residues are labelled.

Since the HE7Q mutation disrupts the endogenous bis-histidyl hexacoordination with HisE7, the comparison of wt Cygb* and its HE7Q mutant provides clues on the transition from exogenous hexacoordination to the endogenous one through the pentacoordinated species. This information is relevant for understanding the properties of the reactive species formed immediately after photolysis of carbonylated Cygb. We recall that the B subunit in the wt Cygb* 1UT0 structure shows an alternative pentacoordinated conformation (estimated at 45% occupancy) [33]. Structural overlays of the HE7Q mutant with bis-histidyl hexa- (subunit A) and pentacoordinated (subunit B) Cygb* yield a rmsd of 0.66 Å and 0.51 Å, respectively. Thus, the overall shape of these structures is very similar. The largest deviations are found at residues 60–66 and 70–83 (CD-D stretch and beginning of helix E; see Figure 4), as the shift from endogenous bis-histidyl hexacoordination to pentacoordination drives the E-helix 1.5 Å away from the distal site pocket (measured on the C_α_ atom of the E7 residue), increasing the distal site volume by about 73 Å^3^. In the pentacoordinated state, HisE7 is still largely accommodated within the distal cavity, oriented towards the heme, but with its Nε atom falling at 4.2 Å from the Fe atom. In the HE7Q mutant the Gln side-chain matches the position of His in the pentacoordinated structure, as the side chain amide N atom is shifted only ∼1 Å relative to the His Nε atom (Figure S2). However, the Gln side chain is pointing to the exterior of the distal site, while the His side chain is oriented to the interior, thus suggesting that the pentacoordinated native Cygb* highlights an intermediate position of HisE7 in the mechanism controlling exogenous ligand binding through competition with the endogenous ligand. Overall, it can be concluded that the D–E region plays a central role in providing the structural degrees of freedom required to switch between endogenous and exogenous hexacoordinated states.

### Molecular dynamics: Structural and dynamical analysis

Extended MD simulations were run to explore the structural integrity and dynamical behavior of *Cygb_h_*, *Cygb_p_* and O_2_Cygb (as noted above, let us remark that two simulation systems were considered for both pentacoordinated and oxygenated states: *Cygb_p_*(HE7Q), *Cygb_p_*(3AG0), O_2_Cygb(HE7Q) and O_2_Cygb(3AG0). For all the simulations the rmsd profiles determined for both the backbone atoms and the heavy atoms were stable along the whole trajectories (see Figure S3).

**Figure 5:**
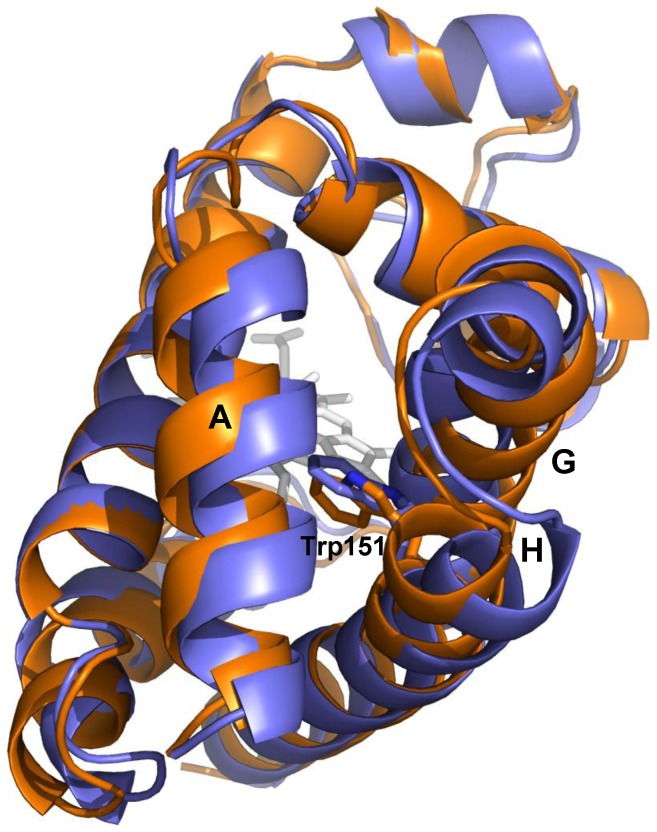
Structural differences in the two species of O_2_Cygb. Superposition of the average structures derived for the two oxygenated forms of Cygb (built up using as templates the X-ray structures of HE7Q mutant and 3AG0, which are shown in violet and orange, respectively). The helices that surround Trp151 are also labelled.

The structural similarity between the different species can be examined from the rmsd between the average structures derived from 10^4^ snapshots collected in the last 10 ns of the trajectories. The results (Table 1) point out a significant difference in the transition from *Cygb_h_* to *Cygb_p_* leading to a rmsd of 1.7 Å, which can be primarily ascribed to the rearrangement of helix E (see above). A slightly larger rmsd (about 2.1 Å) is found between *Cygb_h_* and O_2_Cygb, indicating the occurrence of additional structural rearrangements upon ligand binding. On the other hand, the rmsd between pentacoordinated and O_2_-bound proteins built up using the same template (1.28 and 0.75 Å for simulation systems built up from X-ray structures HE7Q and 3AG0, respectively) is lower than the value determined for the two pentacoordinated (1.55 Å) or the two ligand-bound (1.14 Å) proteins. This finding is surprising, as one would have expected a larger resemblance for Cygb species having the same coordination state. Moreover, this trend suggests that the conformational change associated with Trp151 affects the backbone arrangement, at least in certain structural elements. In fact, inspection of the corresponding structures reveals differences in the arrangement of helices A, G and H (see Figure 5).

**Table 1:** Structural comparison between different coordinated species of Cygb.

	*Cygb_p_*(HE7Q)	*Cygb_p_*(3AG0)	O_2_Cygb(HE7Q)	O_2_Cygb(3AG0)
*Cygb_h_*	1.67	1.83	2.14	2.07
*Cygb_p_*(HE7Q)		1.55	1.28	1.75
*Cygb_p_*(3AG0)			1.23	0.75
O_2_Cygb(HE7Q)				1.14

Rmsd (Å) between energy-minimized average structures derived from 10^4^ snapshots taken along the last 10 ns of trajectories run for bis-histidyl hexacoordinated (*Cygb_h_*), pentacoordinated (*Cygb_p_*(HE7Q), *Cygb_p_*(3AG0)), and oxygenated (O_2_Cygb(HE7Q) and O_2_Cygb(3AG0)) species.

**Figure 6:**
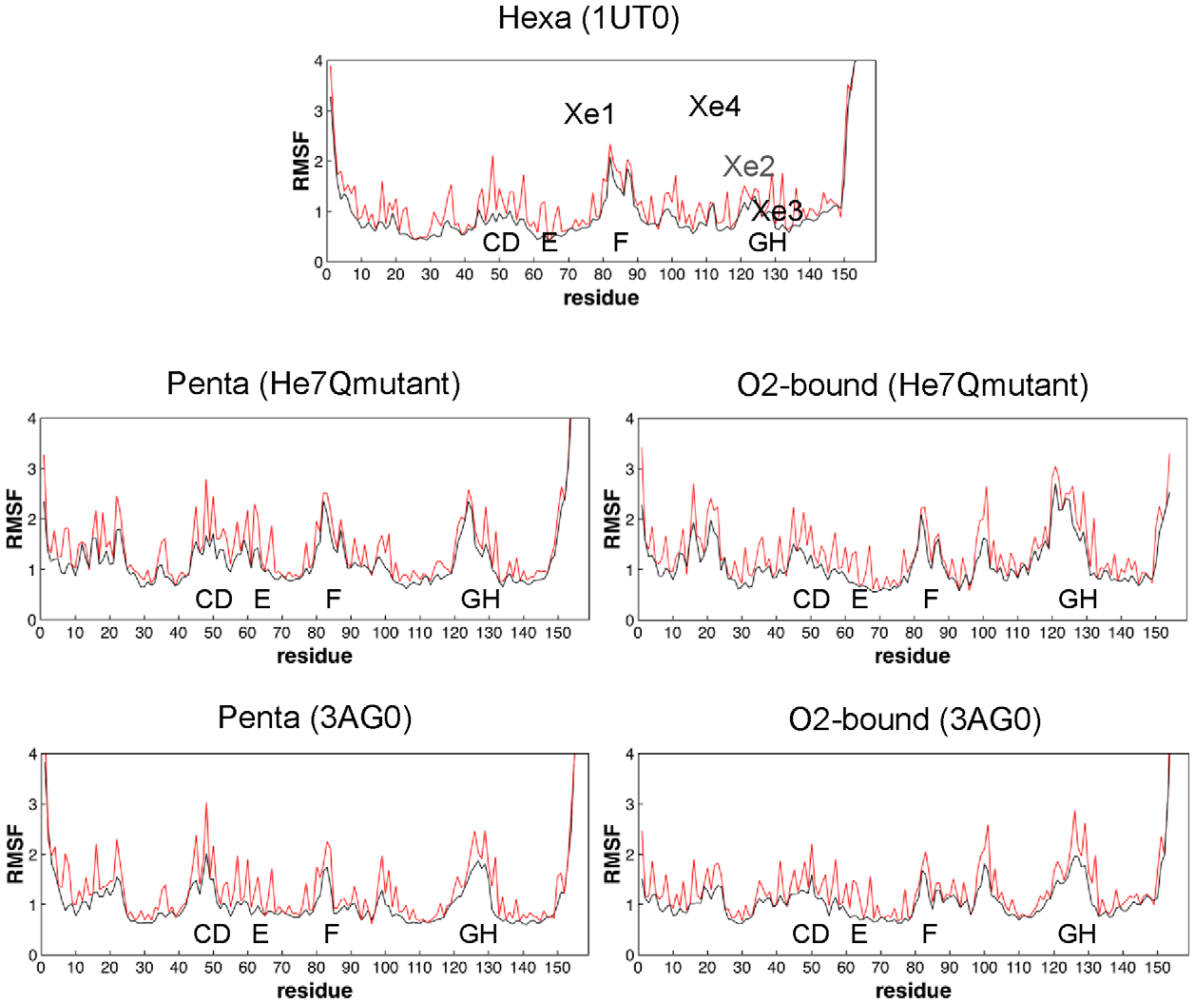
Positional fluctuations of residues. Representation of the root-mean square fluctuations (Å) of residues for the five simulated systems: *Cygb_h_* (from 1UT0), *Cygb_p_* (from HE7Q mutant and 3AG0), and O_2_Cygb (from HE7Q mutant and 3AG0). The positional fluctuations determined for the residue atoms of uniquely the backbone are represented in red and black, respectively.

The root-mean square fluctuation (rmsf) of residues exhibits a similar overall pattern for all the simulated structures, although differential trends can be observed for the distinct simulated species (Figure 6). For *Cygb_h_*, the major fluctuations affect residues in loops CD and helix F. As expected, transition from *Cygb_h_* to *Cygb_p_* enhances the fluctuations, and the most apparent effect is observed in helix E and loop GH, thus reflecting the increased flexibility due to loss of the restraint imposed by the HisE7-heme bond. Finally, coordination of the exogenous ligand reduces the protein fluctuations, especially in loop CD and helix E, though there are enhanced fluctuations in the loops that connect helices F and G, as well as G and H.

Essential dynamics was used to examine the influence of the coordination state on the protein dynamics. Diagonalization of the positional covariance matrix determined for the backbone atoms points out that few motions account for a significant fraction of the structural variance. Thus, about 40% and 65% of the backbone conformational flexibility is accounted for by the first 3 and 10 principal components (Table S6). In *Cygb_h_* the first eigenvector primarily involves the motion of the first half of helix F, the loop EF and the beginning of helix A (see Figure S4). In contrast, the dynamical behavior of *Cygb_p_* is more global, involving the motion of a larger number of structural elements, as expected from the release of the constraint imposed by the HisE7-Fe bond. The most remarkable effect concerns the motion of helix E, which is coupled to deformations in loops CD and EF. Noteworthy, the conformational change of Trp151 introduces differential trends in the flexibility of certain structural elements. Thus, compared to *Cygb_p_* started from HE7Q mutant, the simulation started from 3AG0 shows that helix A is more flexible, while loops GH and EF are more rigid. Finally, in the oxygenated Cygb (started from HE7Q), the major deformation involves loops GH and FG as well as helix G and the last segment of helix A, with minor contributions of the CD and EF loops. Again the Trp151 conformational change introduced in the simulation started from 3AG0 leads to distinct trends, as noted in the enhanced motion of helix H and an increased flexibility in loop EF and helices A and H.

The similarity between the structural fluctuations of the protein backbone was measured by means of the similarity index *ξ_AB_* (Table 2; see also Experimental Methods in Supporting Information S1), which takes into account the nature of the essential motions and their contribution to the structural variance of the protein. Whereas self-similarities vary from 0.70 to 0.79, cross-similarity indexes vary from 0.57–0.67. Thus, though there is a notable overlap between the dynamical motions of the protein skeleton in the different coordination states, there are distinctive trends between bis-histidyl hexacoordinated, pentacoordinated and ligand–bound states, which leads to the gradual transition of protein dynamics from loop EF and segments of helices A and F in *Cygb_h_* to loops CD and helices E and A in *Cygb_p_*, and finally to loop GH and helices A, G and H in the ligand-bound species (see above).

**Table 2:** Self- and cross-similarity indexes determined for the active space of essential motions derived for the different coordinated species of Cygb.

	*Cygb_h_*	*Cygb_p_*(HE7Q)	*Cygb_p_*(3AG0)	O_2_Cygb(HE7Q)	O_2_Cygb(3AG0)
*Cygb_h_*	0.79	0.61	0.57	0.60	0.59
*Cygb_p_*(HE7Q)		0.70	0.59	0.62	0.61
*Cygb_p_*(3AG0)			0.73	0.61	0.65
O_2_Cygb(HE7Q)				0.70	0.66
O_2_Cygb(3AG0)					0.78

Self- similarity indexes were determined by considering the essential motions derived from the snapshots sampled in time windows 20–60 and 60–100 ns in a single trajectory. Cross-similarity indexes were determined by averaging the values obtained from the comparison of the different time windows in two trajectories. The active space comprised 30 eigenvectors, which explain around 85% of the structural variance.

### Analysis of ligand binding sites

Previous X-ray studies have shown that the bis-histidyl hexacoordinated form of Cygb (PDB codes IUX9 and 1URY; [16]) can accommodate up to four Xe atoms in the interior of the protein. The location of the Xe binding sites was predicted by GRID calculations, which identifies energetically favorable regions for placing a Xe atom (Figure S5). The analysis of the Connolly surfaces also revealed a complex and extended system of internal cavities, lined by numerous hydrophobic residues, i.e. Trp31, Leu34, Ile45, Leu46, Met86, Leu89, Val92, Val93, Leu106, Val109, Phe124, Leu127, Ile131, Val134, Val135, Phe139, Trp151, Leu154, Ile158.

**Figure 7:**
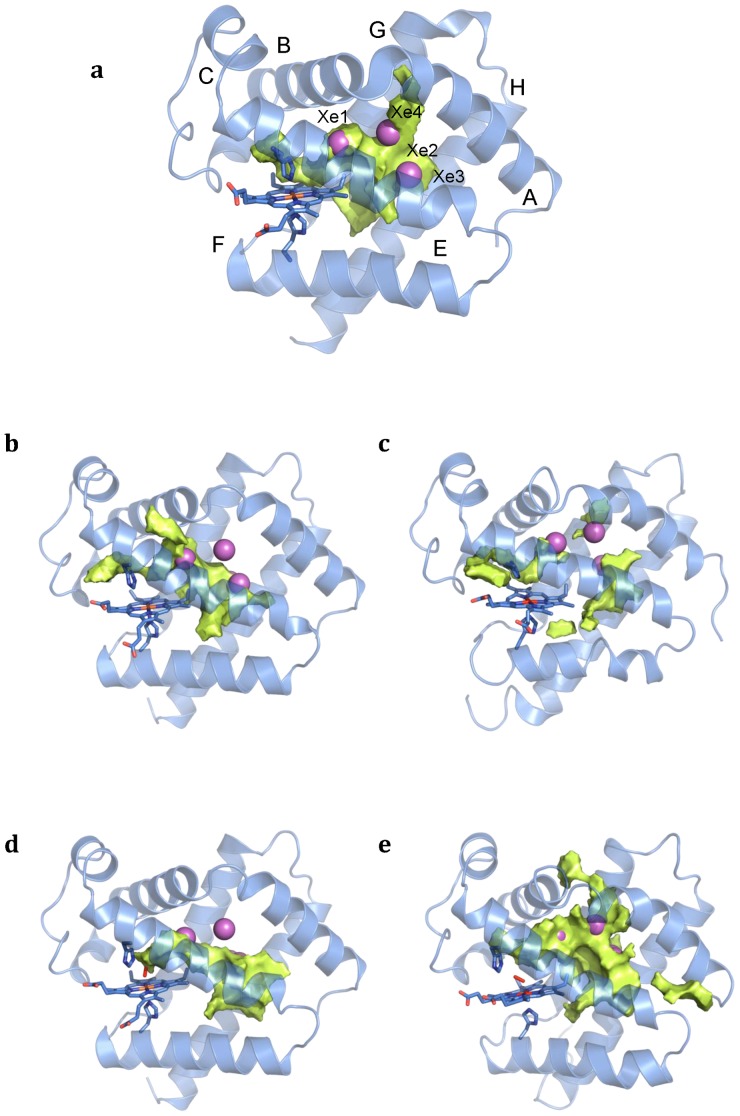
Representation of the average cavities found by MDPOCKET calculations. **a.** *Cygb_h_*; **b.** *Cygb_p_* from HE7Q mutant; **c.** *Cygb_p_* from X-ray 3AG0; **d.** O_2_Cygb from HE7Q mutant; **e.** O_2_Cygb from X-ray 3AG0. The cavities and tunnels detected for the distinct coordination states of the protein are shown in pale yellow. The Xe binding sites found in the X-ray crystallographic structure are numbered and represented as spheres (magenta).

These trends are reflected in the MDpocket analysis of *Cygb_h_* (Figure 7a), which shows a network of interconnected pockets that encompass the four Xe atoms. The results also permit to visualize up to four potential pathways connecting the distal pocket and the solvent, which might provide exchange routes for ligands. Nevertheless, none of them provides a well defined access to the bulk solvent, due to the compactness and reduced flexibility of *Cygb_h_*. One corresponds to the egression by the distal cavity, likely through a His gate mechanism. This pathway, however, is impeded by the fixed orientation of the distal His due to the HisE7-heme bond. Another pathway involves the migration from Xe binding site 4 and passing through a tunnel defined by residues close to the loop AB (Leu34, Val41) and the final segment of helix G (Phe139). A third route connects Xe binding site 2 and the protein surface passing through helices G and H (below the plane of Trp151, and close to Leu132). In fact, the putative role of this pathway is supported by the presence of two water molecules in this area upon inspection of the X-ray structure 1UMO [33]. Finally, the last pathway implies migration via helices E and F, though access to bulk solvent is impeded by the side chains of Val92 and Val105.

Compared to *Cygb_h_*, the analysis of *Cygb_p_* (started from HE7Q mutant) is very different, as three ligand migration pathways can be easily identified (Figure 7b): the His gate pathway from the distal site, the passage through helices E and F, and a new pathway starting from Xe binding site 1 and passing through helices B and E (delimited by residues Val43, Cys83 and Met86). In contrast, at the same isocontour the passage through helices G and H do not permit access to the bulk solvent. When the same analysis is carried out for the simulation started from 3AG0, however, there is a significant reduction in the accessible volume in the interior of the matrix, and only the routes passing through the distal His gate and between helices E and F are clearly defined (Figure 7c).

Finally, the analysis of O_2_Cygb reveals the existence of a big cavity in the interior of the protein. In the simulation started from the HE7Q mutant there is no clear passage to the bulk solvent (Figure 7d). However, in the trajectory started from 3AG0, a well defined pathway that connects the primary docking site to bulk solvent, passing through residues in loop AB and the final segment of helix G, is observed (Figure 7e). The analysis also suggests the potential involvement of another pathway that involves the migration through distinct pocket sites and the exit via a passage located between helix A and loop EF (limited by residues Leu96, Leu154 and Leu157).

The preceding findings point out the plasticity of the internal cavities and exit pathways in Cygb, which are largely influenced not only by the coordination state, but also by the conformation adopted by Trp151. This finding is further corroborated by inspection of the energetically favorable regions for ligand migration derived from ILS calculations (see Figure S6), which show a similar localization of the internal cavities compared to MDpocket analysis.

### NO dioxygenase activity

It is well known that O_2_-bound HbA and Mb react rapidly with NO, yielding nitrate anion. More recently, it was also shown that Ngb catalyzes the same reaction with a comparable efficiency [51]. The efficiency of O_2_-bound Mb to scavenge NO in the heart and skeletal muscle was suggested to protect cytochrome *c* oxidase from being inhibited by NO [52], [53], a role confirmed by *in vivo* experiments carried out with Mb knockout mice [54]. A neuroprotective role, through an NO scavenging activity [51], was put forward for Ngb [14]. Similar to Mb and Ngb, NO detoxification through a NO dioxygenase activity has been proposed as a possible function of Cygb. Central to this action appears to be an efficient gated delivery of reactants, supported by a specific and ligation dependent system of cavities and tunnels, as supported by the analysis of ligand binding kinetics and MD simulations, which highlight a dynamic system of cavities (see above).

**Figure 8:**
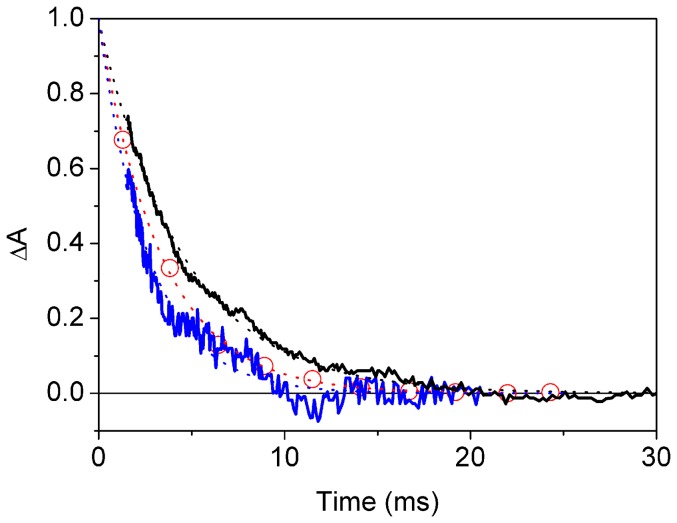
Time course of NO conversion to nitrate anion. Time course of decay of the Fe^3+^ peroxynitrite intermediates to metCygb (3 µM, black solid line) and metHbA (3 µM, grey solid line) after mixing with NO to a final concentration of 9 µM. The reaction progress, displayed in the plot after normalization, was monitored through the absorption changes at 419 nm. For comparison, we also plot the decay of the Fe^3+^ peroxynitrite intermediates to metNgb (open circles) (data from ref [51]). Time courses can be perfectly reproduced with exponential relaxations (dotted lines). Fitted rate constants are 370±10 s^−1^ for Cygb, 300±10 s^−1^ for Ngb, and 220±2 s^−1^ for HbA. T = 20°C.

**Figure 9:**
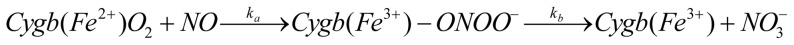
Reaction mechanism for the conversion of NO and O_2_ to nitrate anion [**51**].

To verify the effectiveness of Cygb as an enzyme catalyzing the NO-oxygenase activity, we have thus investigated the reaction of O_2_Cygb with NO *in vitro* using rapid mixing methods. The O_2_Cygb protein can be prepared by exposing an enzymatically reduced protein solution with an air equilibrated buffered solution. Rate constants for oxygen binding to and dissociation from Cygb are 3×10^7^ M^−1^ s^−1^ and 0.3 s^−1^, respectively, and result in an equilibrium constant of 10^6^ M^−1^ [5]. The O_2_-bound Cygb solution was then mixed with a NO solution and the reaction was monitored by following the concomitant absorbance changes. Upon addition of the NO-containing solution, Cygb(Fe^3+^) is readily formed. The spectrum is consistent with that of the ferricyanide-oxidized protein (data not shown). Similar to Ngb, a peroxynitrite intermediate is assumed to be formed within the dead time of the instrument [51], which then decays within a few milliseconds, according to the kinetic scheme in Figure 8. While formation of the peroxynitrite is too fast to be resolved, a lower limit for the rate *k*_a_ can be estimated on the order of 10^8^ M^−1^ s^−1^, in keeping with the previous estimate for Ngb [51]. Figure 9 shows the time course of the decay of Cygb(Fe^3+^)-ONOO^−^, along with the decays of the analogous intermediates for Ngb and HbA. The reaction proceeds with a first order rate constant *k*_b_ = 370±10 s^−1^, a value slightly larger than the one observed for Ngb (300±10 s^−1^) [51], and nearly twice as large as the one observed for HbA (220±2 s^−1^).

## Discussion

The preceding results have shown that the internal cavities of Cygb exhibit a large degree of structural plasticity, which reflects the changes associated with the coordination state of the heme and the conformational flexibility of residues in the inner cavity, such as Trp151. In turn, this trend provides a basis to realize two major experimental findings observed in ligand binding kinetics: i) the differences in the kinetic behavior found for Cygb in solution or encapsulated in gels, and ii) the complexity of the dynamical system of cavities and its impact on ligand rebinding.

### Effect of gel encapsulation on CO rebinding kinetics

The results reveal fundamental differences in the kinetic behaviour of COCygb and Cygb+CO gels, which suggests that distinct structural or dynamical alterations take place in the protein. Thus, the rebinding kinetics to Cygb+CO gels is almost indistinguishable from the signal measured in solution, leading to the formation of the bis-histidyl hexacoordinated species. In contrast, this latter species is absent in the gel containing COCygb, indicating that the pore likely exerts a steric hindrance that prevents the conformational transition to endogenous bis-histidyl hexacoordination. Accordingly, COCygb gels strongly favor the pentacoordinated species, by reducing the equilibrium binding constant for endogenous bis-histidyl hexacoordination (from 83 for Cygb solutions to 0.9 for COCygb gels, as determined from the rate constants in Table S1). Inhibition of the bis-histidyl hexacoordinated species exerted by the gel occurs mostly through enhancement of the dissociation rate constant. By contrast, it is interesting to observe that Cygb+CO gels enhance bis-histidyl hexacoordination, the equilibrium binding constant becoming 170 (Table S2).

The different CO rebinding kinetics to COCygb and Cygb+CO gels can be understood on the basis of the dynamic adaptation of the protein structure in response to different ligation states of the heme (carbonylated, pentacoordinated, or bis-histidyl hexacoordinate). The structure of HE7Q Cygb* shows that the shift from bis-histidyl hexacoordination to pentacoordination is accompanied by a significant displacement of helix E, which expands the volume of the distal cavity. Moreover, the essential dynamics reveals that the main motion involves the bending motion of helices E and F, coupled to deformations in loop CD. Therefore, it is reasonable to foresee that the shape of the pore wrapped by the silica gel around the protein may not impede the structural transition between penta- and bis-histidyl hexacoordinated structures, which implies the displacement of helix E towards the heme. Thus, the structural and dynamical properties of *Cygb_p_* allows us to realize the enhanced bis-histidyl hexacoordination observed in CO rebinding experiments for Cygb+CO gels. In contrast, the constraints imposed by the pores in COCygb gels are much more effective in inhibiting the transition to the bis-histidyl hexacoordinated structure. This effect can be attributed to the specific structural and dynamical properties determined for the ligand-bound species. Thus, the results obtained for O_2_Cygb reveal not only the structural alteration in helices A, G and H, which is particularly relevant after conformational alteration of Trp151 (in the simulation started from 3AG0), but also distinct dynamical motions, which mainly affect loop GH and helices A, G and H, whereas helix E is much less flexible compared to *Cygb_p_*. Therefore, the shape of the silica gel that encloses the protein can be expected to trap the structure of helix E, thus preventing (or at least slowing down) the deformation of helix E towards the heme required to form the bond between HisE7 and the Fe atom.

Overall, the relevant difference found in the rebinding kinetics studies performed for Cygb+CO and COCygb gels, and the larger similarity of the former with the kinetic behaviour measured for the protein in solution can be mainly ascribed to the differential trends in protein dynamics observed for the different coordination states.

### Dynamics of inner cavities and ligand migration

The response of CO rebinding kinetics to environmental parameters reveals a complex kinetic interplay between ligand migration through internal cavities and structural rearrangements, which raises questions about the implications for gating the delivery of reactants to the heme.

**Figure 10:**
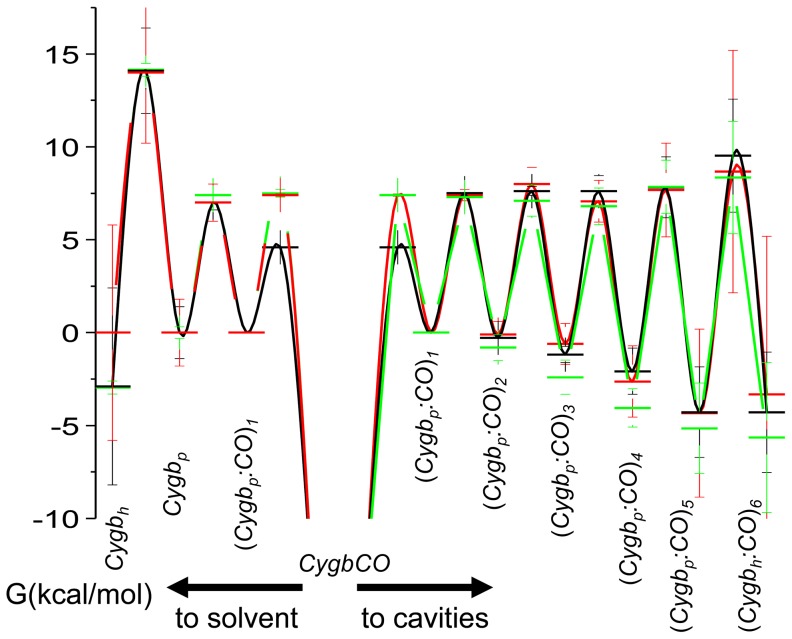
Schematic representation of the energetics of ligand migration in Cygb. Free energy at 20°C for reaction intermediates, estimated from the activation energies for forward and reverse rate constants reported in Table S2, and by arbitrarily setting to 0 the free energy of the state (*Cygb_p_:CO*)_1_. Cygb solution, black lines and symbols; COCygb gels, red lines and symbols; Cygb + CO gels, green lines and symbols.

Activation free energies, determined from the temperature dependence of the forward and reverse rate constants (Tables S3 and S4 in Supporting Information), provide an estimate of the energetic profile encountered by the ligand across its migration path, which is also sensitive to the structural changes imposed by bis-histidyl hexacoordination. Thus, Figure 10 shows that the free energy of the ligand decreases systematically along the migration pathway, with a rather sharp drop for the longer lived (*Cygb_p_:CO*)_5_ and (*Cygb_h_:CO*)_6_ intermediates (see kinetic scheme in Figure 2). Conversely, activation barriers for forward and reverse rate constants steadily increase along the migration path and undergo a sharp increase concomitant with formation of the hexacoordinated species. As can be easily seen in Figure 3A, the last cavity (*Cygb_h_:CO*)_6_ is accessed (dark blue lines) only after binding of the distal HisE7 (green lines) has occurred. Analysis of the rebinding kinetics to the HE7Q mutant shows no evidence of the longer-lived intermediate, as rebinding to pentacoordinated species is complete on shorter time scales. The increase in activation energies observed also for (*Cygb_p_:CO*)_3_ 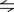
(*Cygb_p_:CO*)_4_ and for (*Cygb_p_:CO*)_4_ 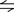
(*Cygb_p_:CO*)_5_ suggests that as yet unidentified conformational changes may play a substantial role.

MDpocket (Figure 7) and ILS (Figure S6) analysis reveals that Cygb exhibits a large structural plasticity that affects both the internal volume and the number and nature of the tunnels depending on the coordination state of the protein, thus leading to distinct migration pathways connecting the distal cavity and the solvent. In the ligand-bound species, two feasible pathways can be envisaged. One pathway connects the primary docking site with an exit channel passing through loop AB and helix G, whereas the other route would involve the ligand egression via helix A and loop EF, though egression through this pathway would require to surpass a larger barrier. In fact, previous computational studies reported that the former pathway was found to be the most feasible route for ligand migration [55]. These findings could explain the observed kinetic results, as in addition to the major escape pathway, which is accessed straight from the distal cavity, exit to the solvent seem to occur from an additional exit point at (*Cygb_p_:CO*)_5_ (≈10%). However, this picture also depends on the conformational change in Trp151, as the results derived for O_2_Cygb starting from HE7Q mutant do not show effective exit pathways (Figure 7). In contrast, *Cygb_p_* presents multiple pathways. The most efficient pathway can be expected to be the His-gate route, proceeding directly to the distal cavity, while the pathway through helices E and F (present in the two simulated forms of *Cygb_p_*) or helices B and E (only found in the structure started from HE7Q) might offer alternative entry points to the protein. Finally, the compact nature of *Cygb_h_* encompasses different pre-formed channels, though not yet clearly accessible to the bulk solvent. Overall, the results clearly indicate an enhanced permeation of the protein upon transition from bis-histidyl hexacoordination to the pentacoordinated state, which should facilitate loading of the heme with a small diatomic ligand. In the ligand-bound state, however, the accessibility seems to be reduced to a single pathway, which could then permit the migration of another ligand to the heme cavity. These features could then be interpreted as a molecular mechanism to ensure a fast binding of O_2_ to *Cygb_p_*, thus rendering the oxygenated protein suitable to capture NO in the ligand-bound state and accomplish in an efficient way the NO dioxygenase (NOD) activity.

This interpretation, however, should be considered with caution, because a simple visual inspection of the time course of the population of the reaction intermediates along the migration pathway for the wt protein in solution (Figure 3A) allows to appreciate the fact that the population of CO docked into internal sites extends well beyond the time scale over which bis-histidyl hexacoordination sets on. Thus, migration is potentially affected by the reshaping of the structure which accompanies the transition from the global fold in the ligand-bound species to the fully relaxed pentacoordinated form, and the subsequent binding of the distal His to the heme. It is worth noting that these events are associated with relevant changes in the dynamical motions of the protein skeleton, as revealed by essential dynamics. Moreover, the ligand accessibility is also affected by the conformation relaxation of Trp151 (see Figure S1). This residue is located in the inner cavity opposite to the heme (the distance from the Cα carbon atom to the heme Fe is around 20 Å) anchored to helix H (position H7) and surrounded by helices A and G. The nitrogen atom of indole ring does not participate in hydrogen bonds with other residues, which justifies the conformational flexibility observed in the X-ray structures. These features provide a basis to explain the impact of the conformational arrangement of Trp151 on the structural reorganization of the protein in the different coordination states, and likely contribute to the different trends observed in the rebinding kinetics in gel (see above). In particular, it might be speculated that the balance between the two conformational states of Trp151 is linked to the coordination state of the protein, so that the transition from endogenous bis-histidyl hexacoordination to the ligand-bound protein and the change in Trp151 act synergistically to regulate ligand migration in Cygb.

In summary, the plasticity of inner cavities and channels may be regarded as a determinant for one of the putative functions suggested for Cygb. Different studies have pointed out that Cygb might be involved in scavenging of NO reactive species [4], [17], [18], [51]. The in vitro experiments reported here suggest that the NOD activity elicited by Cygb indeed occurs at high speed, and thus reactants are provided to and products removed from the active site with high efficiency. The existence of multiple exchange pathways was been demonstrated for Mb, both on experimental [56], [57], [58] and computational [39], [59] grounds. MD simulations also suggested the sequential binding of O_2_ and NO through gating of specific entry tunnels in truncated Hbs [60], [61]. A similar system of cavities was hypothesized to play a relevant role for the NOD activity of Ngb [51]. These cavities were found to be relevant for ligand migration after laser photolysis [21], [47], [48]. The dynamic system of cavities present in Cygb might therefore provide the necessary support to assist the NOD activity. The larger system of cavities of Cygb may be, at least in part, responsible for the observed higher rate for NO dioxygenase activity in comparison to human Hb A and Ngb. Our analysis of *Cygb_p_* offers several pathways for binding of O_2_, though it is reasonable to expect that access will primarily involve the His gate pathway. Alternatively, if the protein skeleton relaxes upon O_2_ binding, NO might access the distal cavity though the channel leading from the bulk solvent (close to loop AB and helix G) to the distal cavity, as found in O_2_Cygb. These processes would lead to a sequential gating of ligands that compares to the one supposed to sustain the NOD activity in TrHb of *Mycobacterium tuberculosis* [60].

## Conclusions

Dramatic changes in CO rebinding kinetics to Cygb are associated to the structural transition from the CO-bound complex (or the deoxy pentacoordinated species) to the bis-histidyl, hexacoordinated species. The conformational transition leads to reshaping of the internal hydrophobic cavities and exit points, and an overall change in the protein dynamics. These findings reflect the significant structural plasticity of Cygb and the strong dependence of the nature and distribution of internal cavities and channels on the coordination state of the proteins. They are also related with conformational changes, such as the rearrangement of Trp151 in the interior of the protein. In turn, these features suggest the existence of a strong linkage between conformational flexibility and biological function of Cygb. In particular, binding of the ligand triggers a series of events, which may be instrumental to sequential processing of diatomic ligands (like e.g. in an NO dioxygenase activity), through gating the exchange of reactants and products along the available exchange pathways. The energetic gating along distinct ligand migration pathways, imposed by the conformational transition between the carbon monoxide complex (or the deoxy pentacoordinated species) and the bis-histidyl, hexacoordinated species, may support sequential substrate entry, characteristic for multisubstrate reactions. Alternatively, the large conformational change induced by ligation of exogenous ligands could be exploited to switch on a signalling state, in keeping with the hypothesized multifunctional role of Cygb.

## Supporting Information

Supporting Information S1 **Experimental methods.** Encapsulation of Cygb and He7Q Cygb*, Kinetic analysis, Crystallization and structural analysis of HE7Q Cygb*, and MD simulations.
(DOC)

Figure S1 **Representation of the two conformations found for the indole ring of Trp151 in different X-ray structures.** The endogenous bis-histidyl hexacoordinated protein (1UT0) is represented in blue and the CO-bound protein (3AG0) in gray.
(TIF)

Figure S2 Close-up of the HE7Q Cygb* distal site. Comparisons of the CD loop and the E-helix as observed in the crystal structures of the HE7Q Cygb*-cyanide complex (gray ribbon) and (left) the Cygb* in the endogenous bis-histidyl hexacoordinated state (1UT0 subunit A, orange ribbon) or (right) Cygb* in the pentacoordinated state (1UT0 subunit B, magenta ribbon). Hydrogen bonds are indicated by dashed lines and relevant residues are labelled. The C-N bond length in cyanide is 1.14 Å and the Fe-C distance is 3.22 Å, with an Fe-C-N angle of 89.1° for subunit A (the corresponding geometrical parameters for subunit B are 1.17 Å, 2.87 Å and 117.1°, respectively). In the absence of any heme-ligand coordination, the orientation of cyanide is essentially dictated by van der Waals contacts to residues Val(E11)85 (3.45 Å for both subunits) and Phe(CD1)60 (3.86 Å and 4.05 Å for chain A and B, respectively), and by a hydrogen bond with the side chain N atom of Gln81(E7) (2.80 and 2.55 Å for subunits A and B). The Gln81(E7) side-chain is oriented toward the solvent region, with the side chain O atom hydrogen bonded to Arg(E10)84. This arrangement frees the heme propionate D, which rotates around 90° relative to the orientation assumed in the native Cygb* (not shown).
(TIF)

Figure S3 **Time evolution of RMSD.** Representation of the time evolution of the RMSD (Å) determined for the backbone (black) and heavy (red) atoms with regard to the corresponding energy minimized structures of the template models used to build up the simulated systems.
(TIF)

Figure S4 **Superimposition of the first (cyan) and last (yellow) frames of the first eigenvector.** **a.** *Cygb_h_*; **b.** *Cygb_p_* from HE7Q mutant; **c.** *Cygb_p_* from X-ray 3AG0; **d.** O_2_Cygb from HE7Q mutant; **e.** O_2_Cygb from X-ray 3AG0.
(TIF)

Figure S5 **Closeup of internal cavities.** Closeup view of the internal cavities network identified in the crystal structure of chain A (a) and chain B (b) of the hexacoordinated form of human cytoglobin treated under Xe gas pressure (1UX9). Xe atoms are represented by cyan spheres, while the red contours correspond to energetically and sterically favourable Xe binding sites as identified by GRID computations. The heme and a few residues regulating the communication between the different docking sites are shown in capped sticks.
(TIF)

Figure S6 **Representation of the average cavities found by ILS calculations.** **a.** hCygb; **b.** pCygb from HE7Q mutant; **c.** pCygb from X-ray 3AG0; **d.** O_2_Cygb from HE7Q mutant; **e.** O_2_Cygb from X-ray 3AG0.
(TIF)

Figure S7 **Representative CO rebinding to COCygb solutions.** Upper panel. CO rebinding kinetics to Cygb solutions equilibrated at 1 atm CO (blue circles 20°C; black circles 40°C) and 0.1 atm CO (cyan circles 20°C; red circles 40°C). Lower panel. Lifetime distributions associated with the rebinding kinetics in the top panel. CO rebinding kinetics shows a remarkable temperature dependence. A clear-cut thermal activation is recognizable in many of the kinetic steps identified in the MEM distribution associated with the rebinding kinetics. For example, in the lower panel the band peaked at 87 µs, at T = 40°C and 1 atm CO (black curve), shifts to 350 µs when CO concentration is reduced tenfold (red curve). Both bands shift to longer lifetimes when temperature is decreased (at 20°C they are peaked at 160 µs, blue curve, and 690 µs, cyan curve, at 1 and 0.1 atm CO, respectively).
(TIF)

Figure S8 **Representative analysis of CO rebinding to COCygb solutions.** Analysis of the CO rebinding kinetics to wt Cygb solutions equilibrated with 1 atm CO (black circles) and 0.1 atm CO (red circles). Left, T = 30°C. Right, T = 40°C. The fits (purple lines) are superimposed to the experimental data (circles). In the figures we have also reported the time course of the other relevant species in the scheme in Figure 2, at 1 atm CO (solid lines) and 0.1 atm CO (dotted lines): (*Cygb_p_:CO*)_1_ (black), (*Cygb_p_:CO*)_2_ (blue), (*Cygb_p_:CO*)_3_ (cyan), (*Cygb_p_:CO*)_4_ (magenta), (*Cygb_p_:CO*)_5_ (yellow), (*Cygb_h_:CO*)_6_ (dark blue), *Cygb_h_* (green), *Cygb_p_* (red).
(TIF)

Figure S9 **Representative analysis of CO rebinding to COCygb gels.** Global analysis of the CO rebinding kinetics to COCygb gels (left 40°C, right 30°C) equilibrated with 1 atm CO (black circles) and 0.1 atm CO (red circles). The fits (purple lines) are superimposed to the experimental data (circles). In the figures we have also reported the time course of the other relevant species in the scheme in Figure 2, at 1 atm CO (solid lines) and 0.1 atm CO (dotted lines): (*Cygb_p_:CO*)_1_ (black), (*Cygb_p_:CO*)_2_ (blue), (*Cygb_p_:CO*)_3_ (cyan), (*Cygb_p_:CO*)_4_ (magenta), (*Cygb_p_:CO*)_5_ (yellow), (*Cygb_h_:CO*)_6_ (dark blue), *Cygb_h_* (green), *Cygb_p_* (red).
(TIF)

Figure S10 **Representative analysis of CO rebinding to Cygb+CO gels.** Global analysis of the CO rebinding kinetics to Cygb+CO gels (T = 40°C) equilibrated with 1 atm CO (black circles) and 0.1 atm CO (red circles). The fits (purple lines) are superimposed to the experimental data (circles). In the figures we have also reported the time course of the other relevant species in the scheme in Figure 2, at 1 atm CO (solid lines) and 0.1 atm CO (dotted lines): (*Cygb_p_:CO*)_1_ (black), (*Cygb_p_:CO*)_2_ (blue), (*Cygb_p_:CO*)_3_ (cyan), (*Cygb_p_:CO*)_4_ (magenta), (*Cygb_p_:CO*)_5_ (yellow), (*Cygb_h_:CO*)_6_ (dark blue), *Cygb_h_* (green), *Cygb_p_* (red).
(TIF)

Figure S11 Representative analysis of CO rebinding to HE7Q Cygb* solutions. Global analysis of the CO rebinding kinetics to HE7Q Cygb* solutions at T = 10°C (left) and T = 40°C (right), equilibrated with 1 atm CO (black circles) and 0.1 atm CO (red circles). The fits (purple lines) are superimposed to the experimental data (circles). In the figures we have also reported the time course of the other relevant species in the scheme in Figure 2, at 1 atm CO (solid lines) and 0.1 atm CO (dotted lines): (*Cygb_p_:CO*)_1_ (black), (*Cygb_p_:CO*)_2_ (blue), (*Cygb_p_:CO*)_3_ (cyan), (*Cygb_p_:CO*)_4_ (magenta), (*Cygb_p_:CO*)_5_ (yellow), (*Cygb_h_:CO*)_6_ (dark blue), *Cygb_h_* (green), *Cygb_p_* (red). Analysis of the CO rebinding kinetics to HE7Q Cygb* solutions (Figure S10) shows partly inhibited migration pattern through internal hydrophobic cavities in comparison to the one observed for Cygb solutions. The source for this can be found in the higher reactivity of the this mutant (see Table S4). The kinetics at 1 and 0.1 atm CO can be perfectly reproduced over the whole investigated temperature range.
(TIF)

Table S1 **Microscopic rate constants for Cygb from the fit of the flash photolysis data, at 20°C.** Activation enthalpies and entropies were estimated from the linear Eyring plots for each rate constant *k*_i_ in the temperature range 10–40°C.
(DOCX)

Table S2 **Comparison between microscopic rate constants for wt Cygb solutions, COCygb gels and Cygb+CO gels from the fit of the flash photolysis data, at 20°C.** Activation enthalpies and entropies were estimated from the linear Eyring plots for each rate constant *k*_i_ in the temperature range 10–40°C.
(DOCX)

Table S3 **Activation free energies at 20°C for Cygb solutions and gels.**
(DOCX)

Table S4 Microscopic rate constants for HE7Q Cygb* solutions from the fit of the flash photolysis data, at 20°C. Activation enthalpies and entropies were estimated from the linear Eyring plots for each rate constant *k*_i_ in the temperature range 10–40°C.
(DOCX)

Table S5 Data collection and refinement statistics for HE7Q Cygb*.
(DOCX)

Table S6 **Contribution (%) of the first 10 eigenvectors derived from essential dynamics to the conformational flexibility of the protein backbone.**
(DOCX)
