# Supplementary material for: CO Rebinding Kinetics and Molecular Dynamics Simulations Highlight Dynamic Regulation of Internal Cavities in Human Cytoglobin
Source: PLoS One. 2013 Jan 4;8(1):e49770. doi: 10.1371/journal.pone.0049770 (PMC3537629; doi:10.1371/journal.pone.0049770)
Supplement: Supporting Information S1 — Experimental methods. Encapsulation of Cygb and He7Q Cygb*, Kinetic analysis, Crystallization and structural analysis of HE7Q Cygb*, and MD simulations. (DOC) [file pone.0049770.s001.doc]

**Experimental methods**

***Encapsulation of Cygb and He7Q Cygb****

The encapsulation of Cygb and HE7Q Cygb* in silica gels was carried out following a previously described protocol [1]. A solution containing tetramethyl orthosilicate, water and hydrochloric acid was sonicated for 20 min at 4 °C. An equal volume of a deoxygenated solution containing 10 mM phosphate, pH 6.0, was then added to the sol, which was further deoxygenated for 40 min at 4 °C under flux of nitrogen. Finally, a 200 μM solution of protein in a 50 mM phosphate buffer, pH 7.2, equilibrated with CO at 1 atm, was anaerobically added to the sol. The resulting mixture was layered on quartz plates under anaerobic conditions. Gelation occurred in approximately 20 minutes. The silica wet gels were anaerobically stored in a buffer containing 100 mM phosphate, 1 mM EDTA, 5 mM sodium dithionite, pH 7.0. Before experiments, the gels were soaked in a solution previously equilibrated with CO.

***Kinetic analysis***

In order to highlight the number and the relative roles of the different kinetics phases, the lifetime distributions associated with the observed kinetics were evaluated using the program MemExp (version 3.0) written by P.J. Steinbach [2,3]. MemExp uses the Maximum Entropy Method (MEM) and either nonlinear least squares (NLS) or maximum likelihood (ML) fitting to analyze a general time-dependent signal in terms of distributed and discrete lifetimes. The quality of the fits was always excellent, with the residuals randomly oscillating around zero.

The coupled differential equations associated with the kinetic mechanism of ligand migration were solved numerically and the rate constants were optimized to obtain a best fit to the experimental data. Numerical solutions were determined by using the function ODE15s within Matlab 7.0 (The MathWorks, Inc.). Fitting of the numerical solution to experimental data (and optimization of microscopic rate constants) was obtained with a Matlab version of the optimization package Minuit (CERN).

***Crystallization and structural analysis of HE7Q Cygb****

The protein solution, at 12 mg/ml concentration, was equilibrated at 277 K against a precipitant solution containing 18% (w/v) PEG 4000, 0.2 M NaCl, 0.05 M sodium acetate (pH 4.1–4.6), 0.01 M potassium ferricyanide and 1 mM KCN. They were transferred to the same solution containing a higher concentration of PEG 4000 (22% w/v) and supplemented with 25% (v/v) glycerol, immediately prior to cryo-cooling and data collection at 100 K. All collected data were reduced and scaled using MOSFLM and SCALA, respectively [4 ,5].

The crystal structure of the native Cygb* (PDB code 1UT0) [6] was used as initial model, with the 76–96 aminoacid region deleted (corresponding to the E-helix, which contains the HE7Q mutation). Several cycles of manual rebuilding, using the program Coot [7], and refinement, using the program REFMAC [8] (rigid body and TLS restrained refinement), were carried out to improve the electron density map, and to build the protein region deleted in the original model. The program Procheck [9] was used to assess the stereochemical quality of the protein structure. Atomic coordinates and structure factors have been deposited with the Protein Data Bank (PDB-code 4b3w) [10].

***MD simulations***

Due to the lack of detailed structural information about the N- and C-terminal regions, which are presumably disordered, simulations were performed for the protein core (residues 20-168). The most adequate protonation state at physiological pH was assigned to ionizable residues. The distal His(E7) was protonated on Nδ when coordinated to the heme into *Cygb_h_*, and on Nε in *Cygb_p_* and O_2_Cygb. The starting structures were immersed in a preequilibrated octahedral box of around 8400 TIP3P [11] water molecules, and sodium ions were added to keep electrical neutrality of the simulated systems. The heme parameters were taken from previous works [12,13]. The SHAKE algorithm [14] was used to keep bonds involving hydrogen atoms at their equilibrium length, in conjunction with a 1 fs time step for integration of Newton’s equations of motion. Trajectories were collected in the NPT (1 atm, 298 K) ensemble using periodic boundary conditions and Ewald sums (grid spacing of 1 Å) for long-range electrostatic interactions [15]. The systems were minimized using a multistep protocol, involving first the adjustment of hydrogens, then the refinement of water molecules, and finally the minimization of the whole system [12]. The equilibration was performed by heating from 100 to 298 K in four 50-ps steps at 150, 200, 250 and 298 K. Finally, for each simulated system 100 ns production trajectories were run, collecting frames at 1 ps intervals.

***Similarity index***

The global similarity between two sets of essential eigenvectors is determined using the following equation:


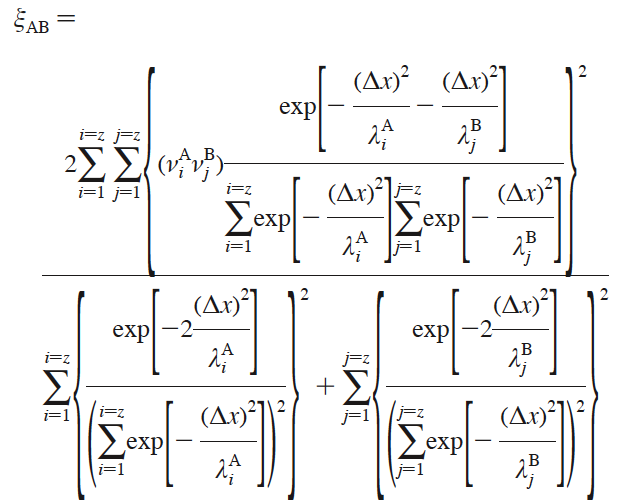


where $\lambda_{i}$ is the eigenvalue (in Å^2^) associated with eigenvector $\mu_{i}$, whose unitary vector is $\nu_{i}$ [16].

**References**

1. Bettati S, Mozzarelli A (1997) T state hemoglobin binds oxygen noncooperatively with allosteric effects of protons, inositol hexaphosphate and chloride. Journal of Biological Chemistry 272: 32050-32055.

2. Steinbach PJ (2002) Inferring Lifetime Distributions from Kinetics by Maximizing Entropy Using a Bootstrapped Model. J Chem Inf Comput Sci 42: 1476-1478.

3. Steinbach PJ, Ionescu R, Matthews CR (2002) Analysis of Kinetics Using a Hybrid Maximum-Entropy/Nonlinear-Least-Squares Method: Application to Protein Folding. Biophysical Journal 82: 2244-2255.

4. Leslie AGM (2003) MOSFLM User Guide, Mosflm Version 6.2.3,. Cambridge, UK: MRC Laboratory of Molecular Biology.

5. Evans PR (1993) Proceedings of the CCP4 study weekend on data collection and processing. CLRC Daresbury Laboratory, UK.

6. deSanctis D, Dewilde S, Pesce A, Moens L, Ascenzi P, et al. (2004) Crystal Structure of Cytoglobin: The Fourth Globin Type Discovered in Man Displays Heme Hexa-coordination. Journal of Molecular Biology 336: 917–927.

7. Emsley P, Cowtan K (2004) Coot: model-building tools for molecular graphics. Acta Crystallographica Sect D 60: 2126-2132.

8. Murshudov GN, Vagin AA, Dodson EJ (1997) Refinement of macromolecular structures by the maximum-likelihood method. Acta Crystallographica Sect D 53: 240-255.

9. Laskowski R, MacArthur M, Moss D, Thornton J (1993) PROCHECK, a program to check the stereochemical quality of protein structure. Journal Applied Crystallography 26: 283-291.

10. Berman HM, Westbrook J, Feng Z, Gilliland G, Bhat TN, et al. (2000) The Protein Data Bank. Nucleic Acids Research 28: 235-242.

11. Jorgensen WL, Chandrasekhar J, Madura JD, Impey RW, Klein ML (1983) Comparison of simple potential functions for simulating liquid water. Journal of Chemical Physics 79: 926−935.

12. Bidon-Chanal A, Martí MA, Crespo A, Milani M, Orozco M, et al. (2006) Ligand-induced dynamical regulation of NO conversion in *Mycobacterium tuberculosis* truncated-hemoglobin-N. Proteins 64: 457-464.

13. Martí MA, Crespo A, Capece L, Boechi L, Bikiel DE, et al. (2006) Dioxygen affinity in heme proteins investigated by computer simulation. Journal of Inorganic Biochemistry 100: 761-770.

14. Ryckaert J, Ciccotti G, Berendsen H (1977) Numerical integration of the Cartesian equations of motion of a system with constraints: Molecular dynamics of n-alkanes. Journal of Computational Physics 23: 327-341.

15. Darden T, York D, Pederson L (1993) Particle mesh Ewald: An N log(N) method for ewald sums in large systems. Journal of Chemical Physics 98: 10089-10092.

16. Perez A, Blas JR, Rueda M, Lopez-Bes JM, de la Cruz X, et al. (2005) Exploring the essential dynamics of B-DNA. Journal of Chemical Theory and Computation 1: 790-800.
