# Supplementary figures and images for: CO Rebinding Kinetics and Molecular Dynamics Simulations Highlight Dynamic Regulation of Internal Cavities in Human Cytoglobin
Source: PLoS One. 2013 Jan 4;8(1):e49770. doi: 10.1371/journal.pone.0049770 (PMC3537629; doi:10.1371/journal.pone.0049770)

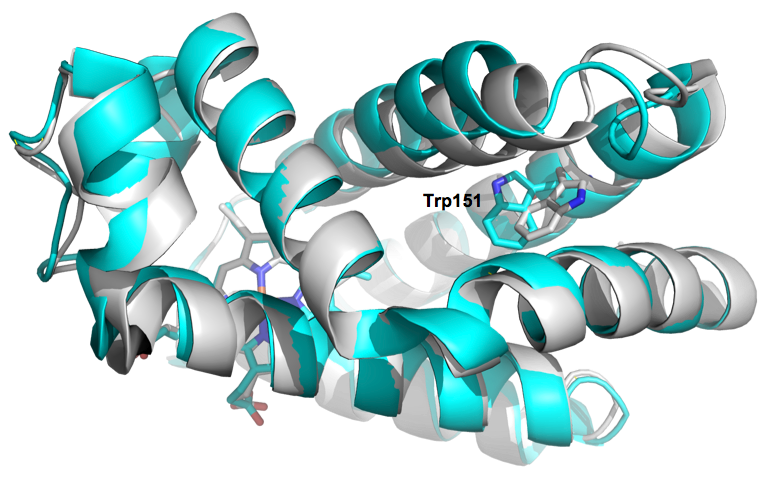

Supplement: Figure S1 — Representation of the two conformations found for the indole ring of Trp151 in different X-ray structures. The endogenous bis-histidyl hexacoordinated protein (1UT0) is represented in blue and the CO-bound protein (3AG0) in gray. (TIF) [file pone.0049770.s002.tif]

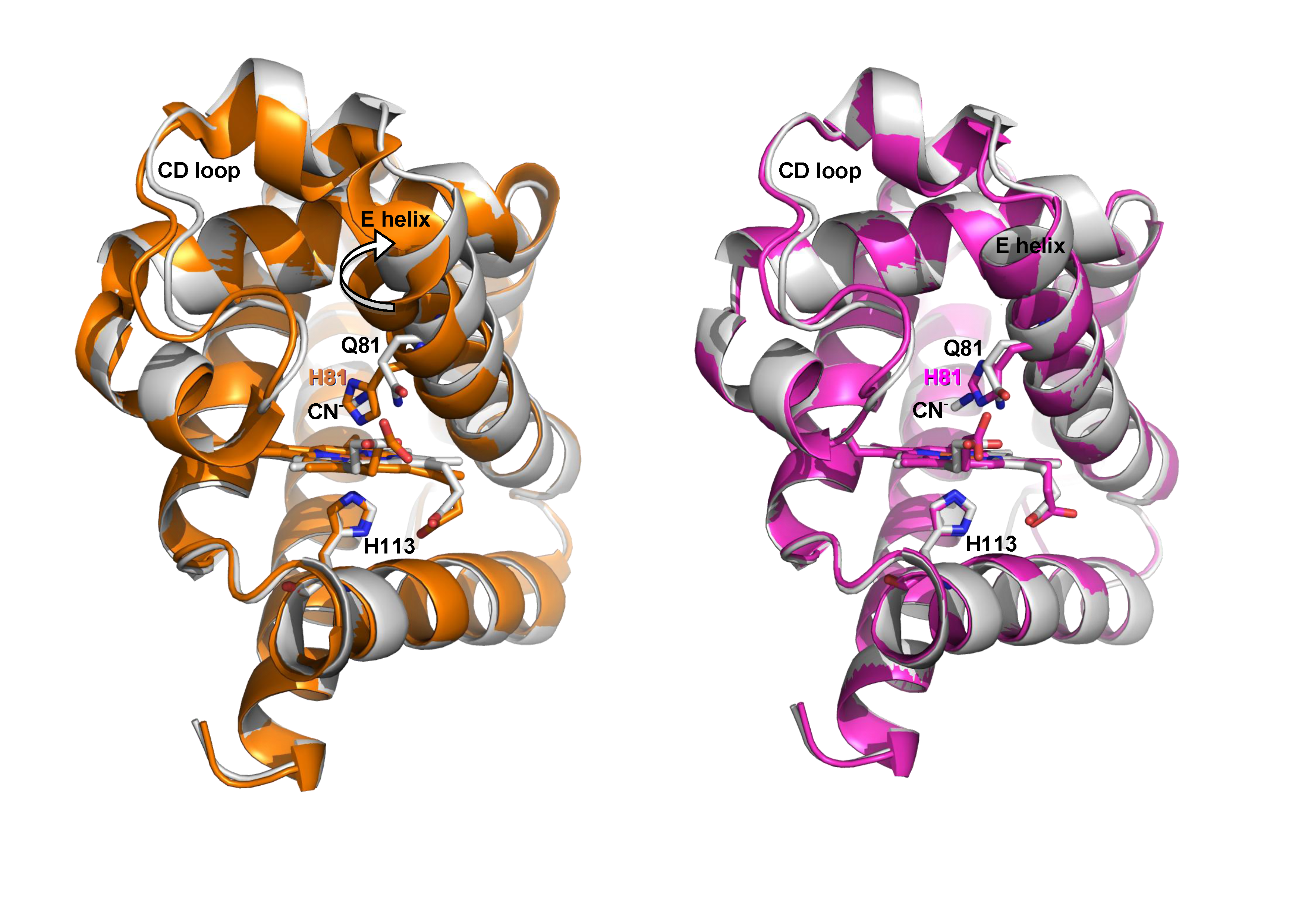

Supplement: Figure S2 — Close-up of the HE7Q Cygb* distal site. Comparisons of the CD loop and the E-helix as observed in the crystal structures of the HE7Q Cygb*-cyanide complex (gray ribbon) and (left) the Cygb* in the endogenous bis-histidyl hexacoordinated state (1UT0 subunit A, orange ribbon) or (right) Cygb* in the pentacoordinated state (1UT0 subunit B, magenta ribbon). Hydrogen bonds are indicated by dashed lines and relevant residues are labelled. The C-N bond length in cyanide is 1.14 Å and the Fe-C distance is 3.22 Å, with an Fe-C-N angle of 89.1° for subunit A (the corresponding geometrical parameters for subunit B are 1.17 Å, 2.87 Å and 117.1°, respectively). In the absence of any heme-ligand coordination, the orientation of cyanide is essentially dictated by van der Waals contacts to residues Val(E11)85 (3.45 Å for both subunits) and Phe(CD1)60 (3.86 Å and 4.05 Å for chain A and B, respectively), and by a hydrogen bond with the side chain N atom of Gln81(E7) (2.80 and 2.55 Å for subunits A and B). The Gln81(E7) side-chain is oriented toward the solvent region, with the side chain O atom hydrogen bonded to Arg(E10)84. This arrangement frees the heme propionate D, which rotates around 90° relative to the orientation assumed in the native Cygb* (not shown). (TIF) [file pone.0049770.s003.tif]

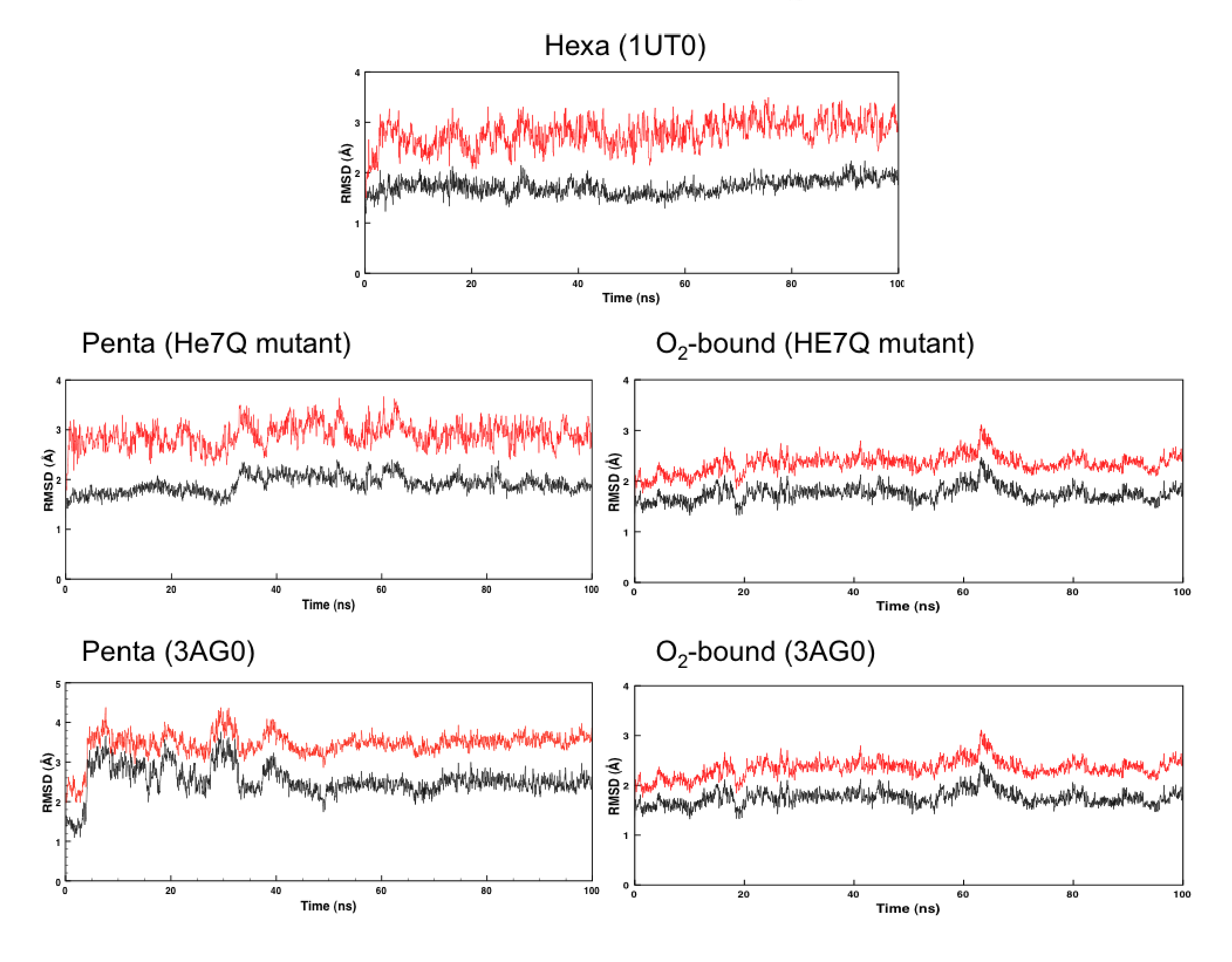

Supplement: Figure S3 — Time evolution of RMSD. Representation of the time evolution of the RMSD (Å) determined for the backbone (black) and heavy (red) atoms with regard to the corresponding energy minimized structures of the template models used to build up the simulated systems. (TIF) [file pone.0049770.s004.tif]

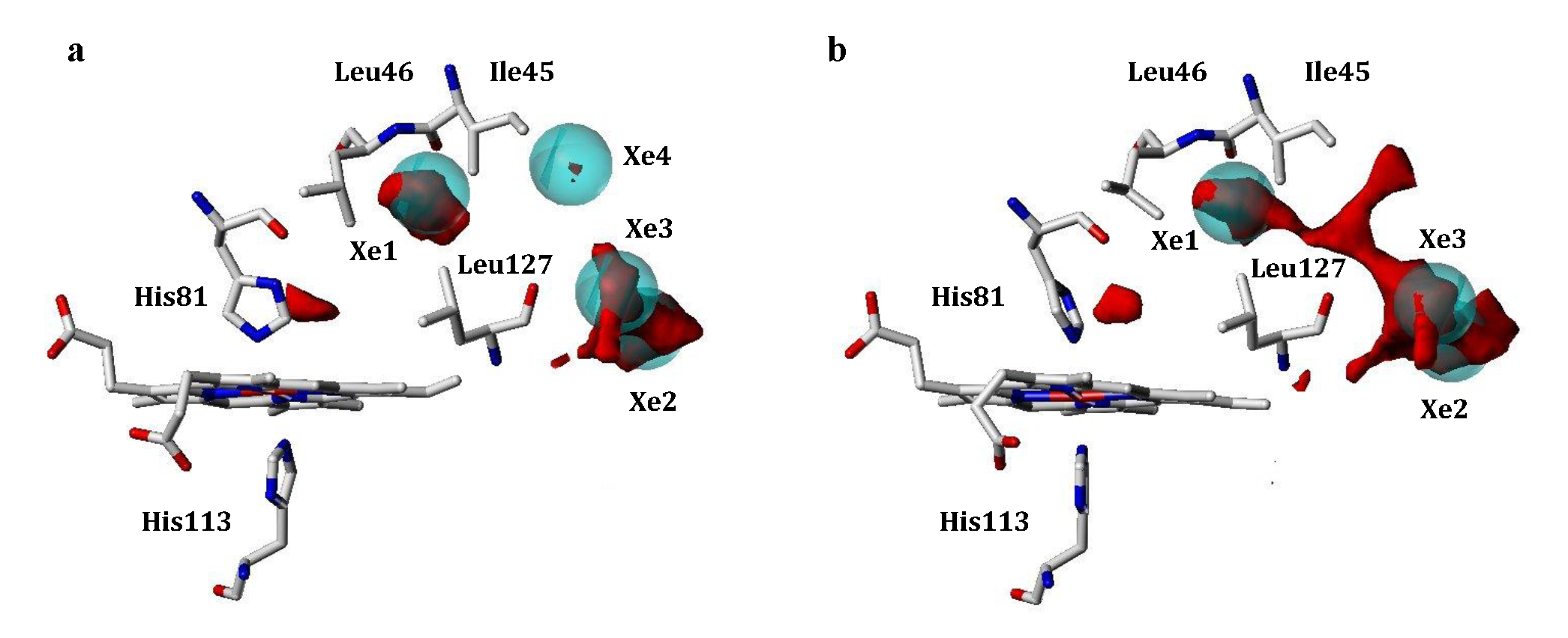

Supplement: Figure S4 — Superimposition of the first (cyan) and last (yellow) frames of the first eigenvector. a. Cygbh; b. Cygbp from HE7Q mutant; c. Cygbp from X-ray 3AG0; d. O2Cygb from HE7Q mutant; e. O2Cygb from X-ray 3AG0. (TIF) [file pone.0049770.s005.tif]

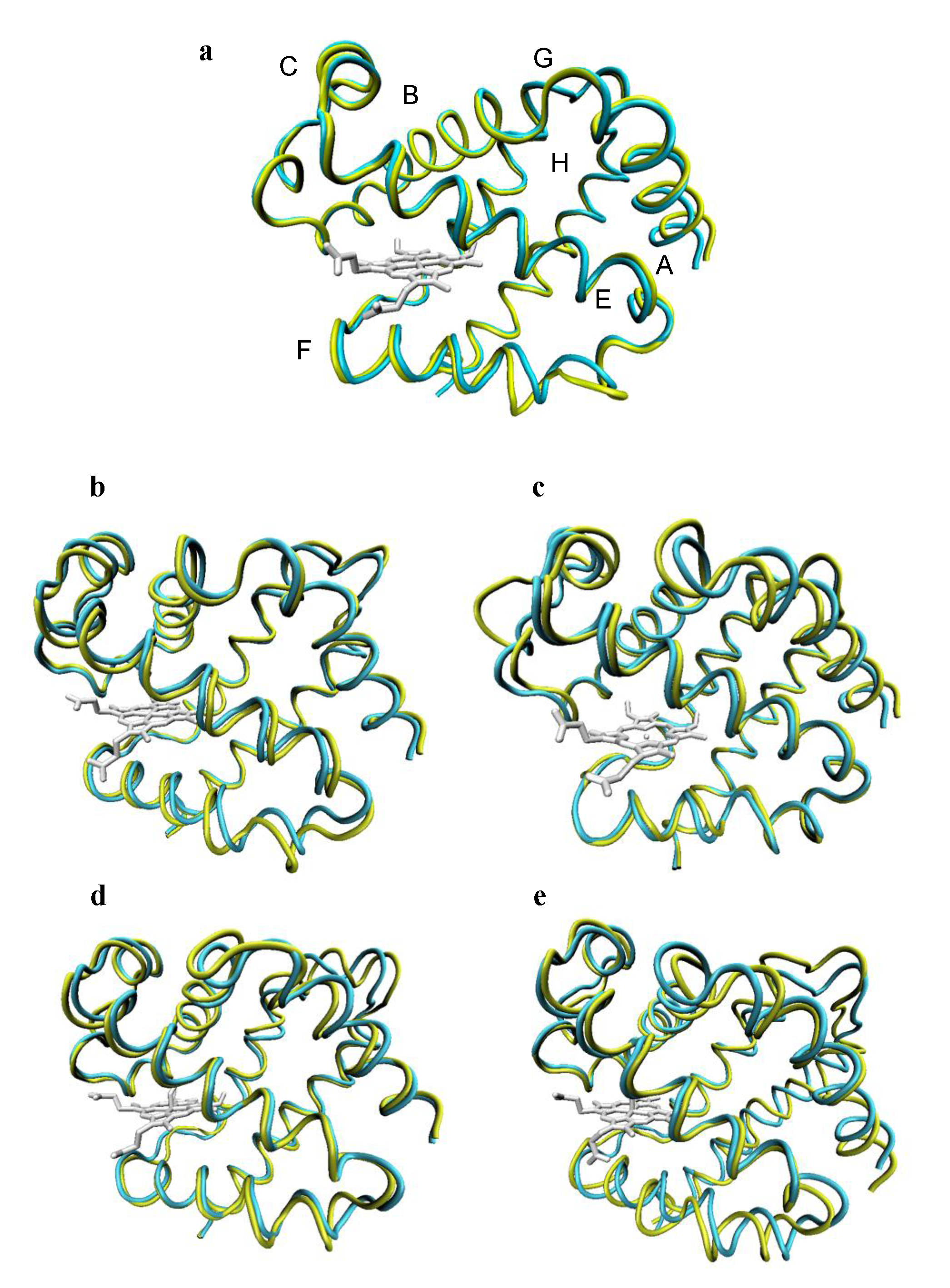

Supplement: Figure S5 — Closeup of internal cavities. Closeup view of the internal cavities network identified in the crystal structure of chain A (a) and chain B (b) of the hexacoordinated form of human cytoglobin treated under Xe gas pressure (1UX9). Xe atoms are represented by cyan spheres, while the red contours correspond to energetically and sterically favourable Xe binding sites as identified by GRID computations. The heme and a few residues regulating the communication between the different docking sites are shown in capped sticks. (TIF) [file pone.0049770.s006.tif]

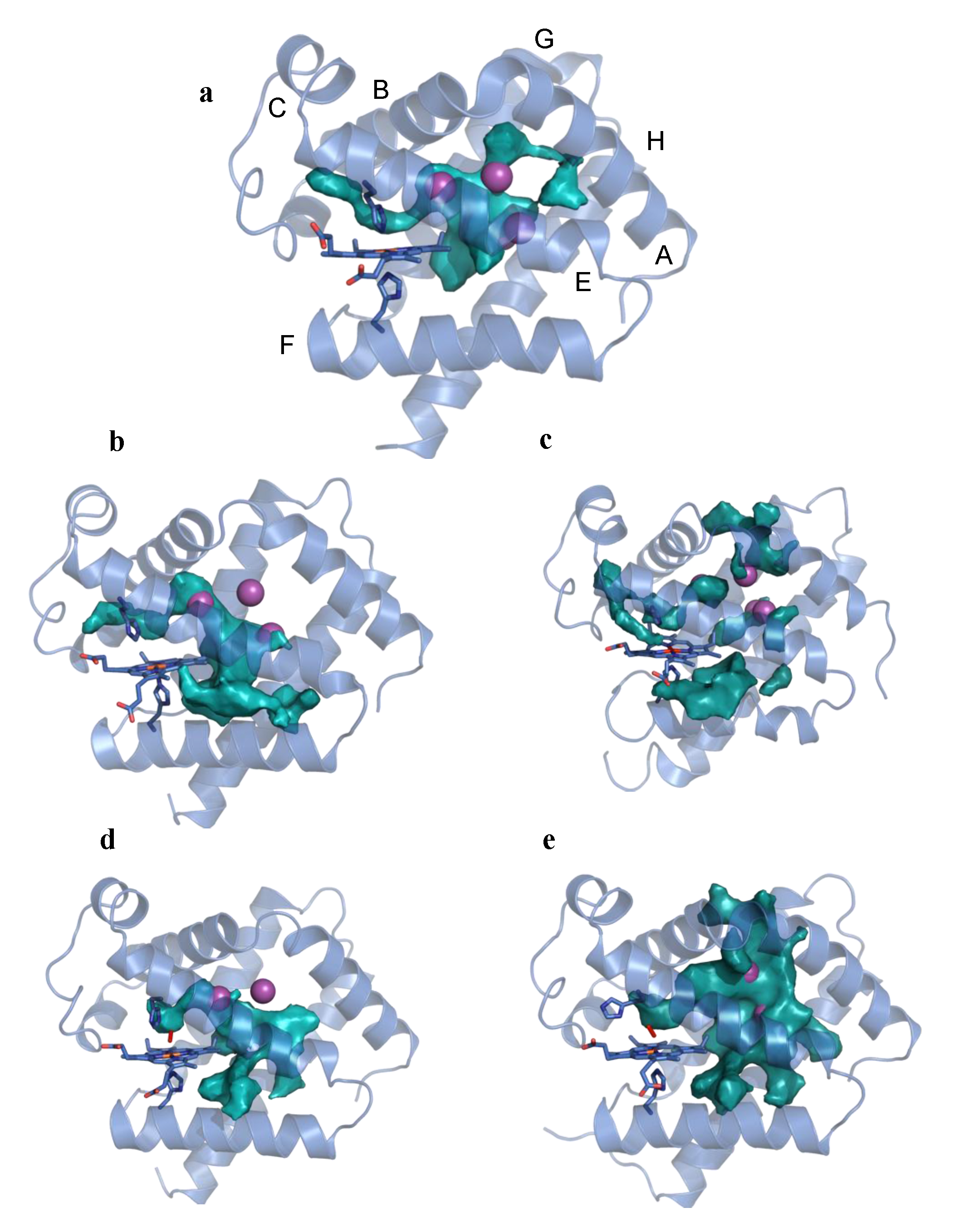

Supplement: Figure S6 — Representation of the average cavities found by ILS calculations. a. hCygb; b. pCygb from HE7Q mutant; c. pCygb from X-ray 3AG0; d. O2Cygb from HE7Q mutant; e. O2Cygb from X-ray 3AG0. (TIF) [file pone.0049770.s007.tif]

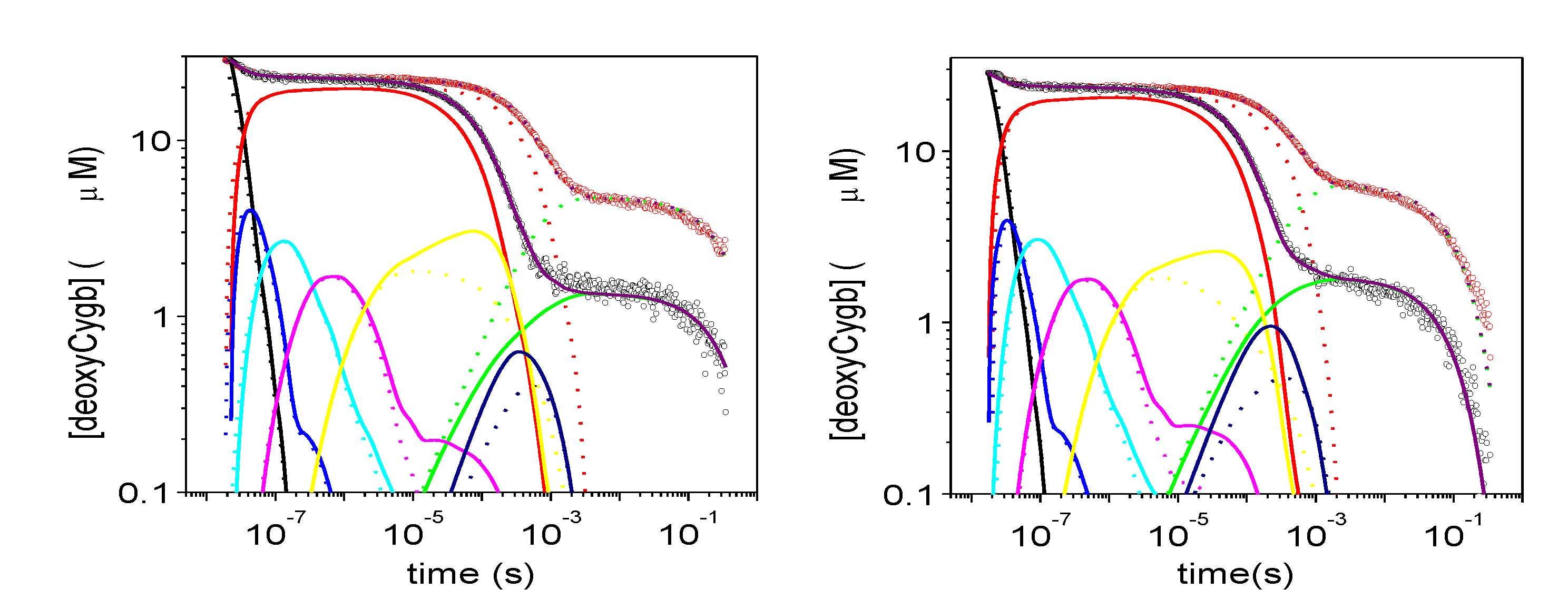

Supplement: Figure S8 — Representative analysis of CO rebinding to COCygb solutions. Analysis of the CO rebinding kinetics to wt Cygb solutions equilibrated with 1 atm CO (black circles) and 0.1 atm CO (red circles). Left, T = 30°C. Right, T = 40°C. The fits (purple lines) are superimposed to the experimental data (circles). In the figures we have also reported the time course of the other relevant species in the scheme in Figure 2, at 1 atm CO (solid lines) and 0.1 atm CO (dotted lines): (Cygbp:CO)1 (black), (Cygbp:CO)2 (blue), (Cygbp:CO)3 (cyan), (Cygbp:CO)4 (magenta), (Cygbp:CO)5 (yellow), (Cygbh:CO)6 (dark blue), Cygbh (green), Cygbp (red). (TIF) [file pone.0049770.s009.tif]

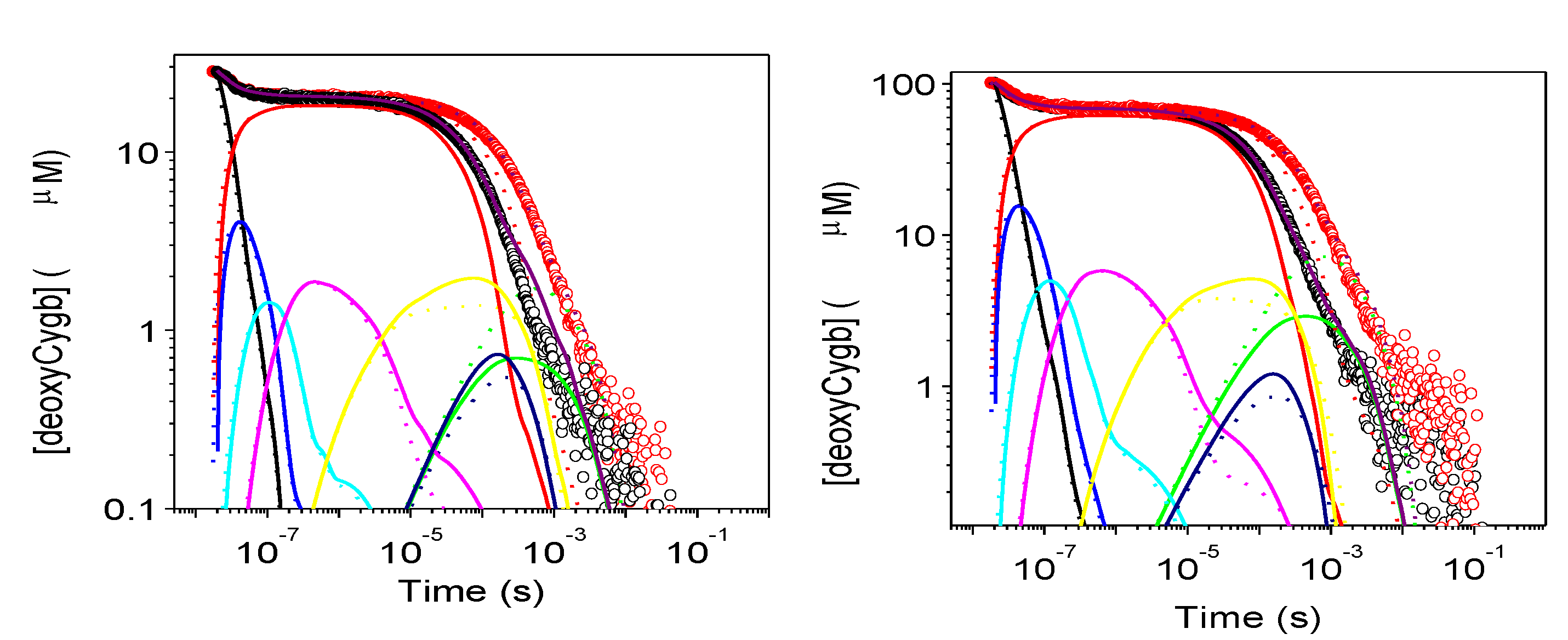

Supplement: Figure S9 — Representative analysis of CO rebinding to COCygb gels. Global analysis of the CO rebinding kinetics to COCygb gels (left 40°C, right 30°C) equilibrated with 1 atm CO (black circles) and 0.1 atm CO (red circles). The fits (purple lines) are superimposed to the experimental data (circles). In the figures we have also reported the time course of the other relevant species in the scheme in Figure 2, at 1 atm CO (solid lines) and 0.1 atm CO (dotted lines): (Cygbp:CO)1 (black), (Cygbp:CO)2 (blue), (Cygbp:CO)3 (cyan), (Cygbp:CO)4 (magenta), (Cygbp:CO)5 (yellow), (Cygbh:CO)6 (dark blue), Cygbh (green), Cygbp (red). (TIF) [file pone.0049770.s010.tif]

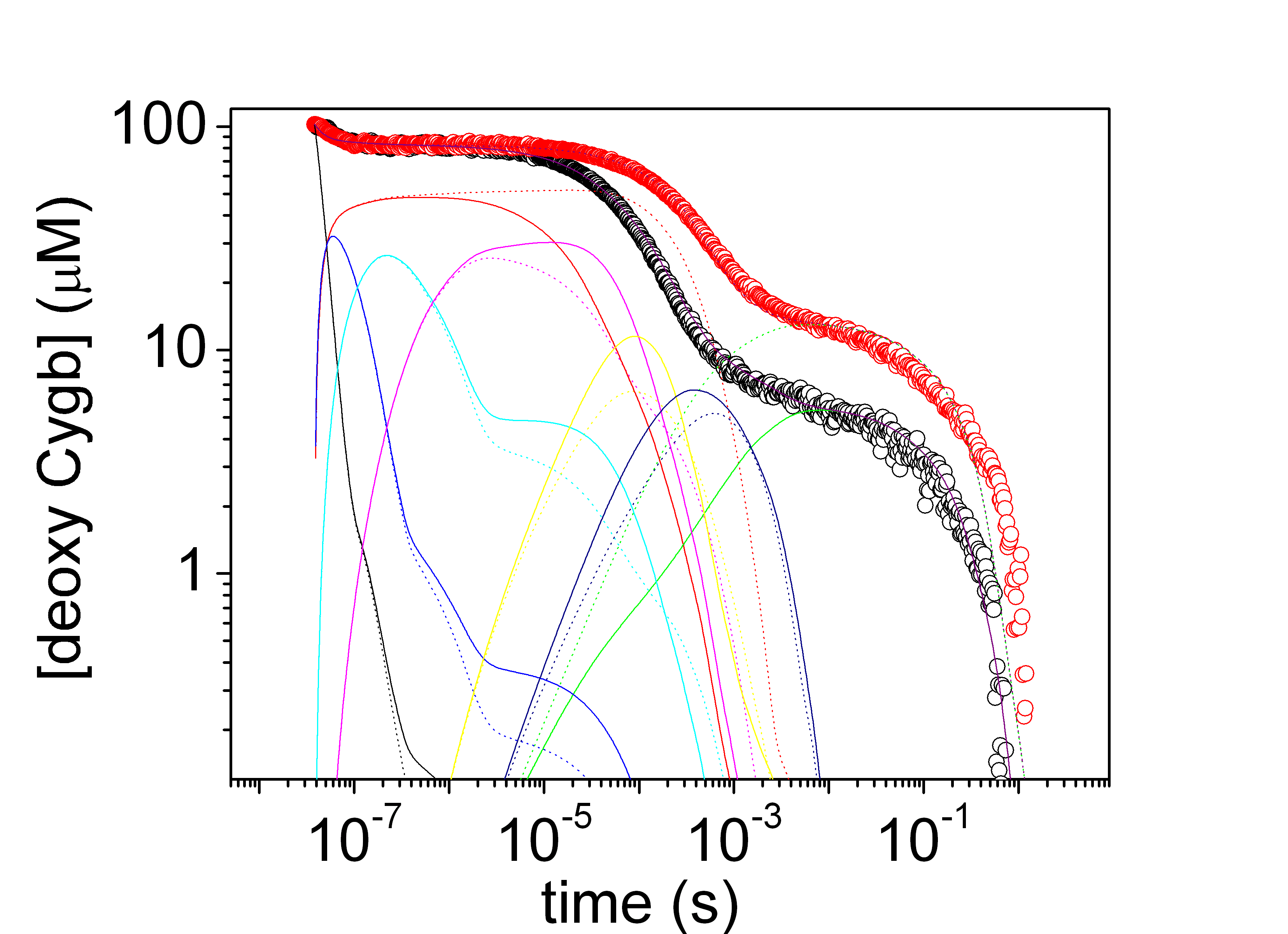

Supplement: Figure S10 — Representative analysis of CO rebinding to Cygb+CO gels. Global analysis of the CO rebinding kinetics to Cygb+CO gels (T = 40°C) equilibrated with 1 atm CO (black circles) and 0.1 atm CO (red circles). The fits (purple lines) are superimposed to the experimental data (circles). In the figures we have also reported the time course of the other relevant species in the scheme in Figure 2, at 1 atm CO (solid lines) and 0.1 atm CO (dotted lines): (Cygbp:CO)1 (black), (Cygbp:CO)2 (blue), (Cygbp:CO)3 (cyan), (Cygbp:CO)4 (magenta), (Cygbp:CO)5 (yellow), (Cygbh:CO)6 (dark blue), Cygbh (green), Cygbp (red). (TIF) [file pone.0049770.s011.tif]

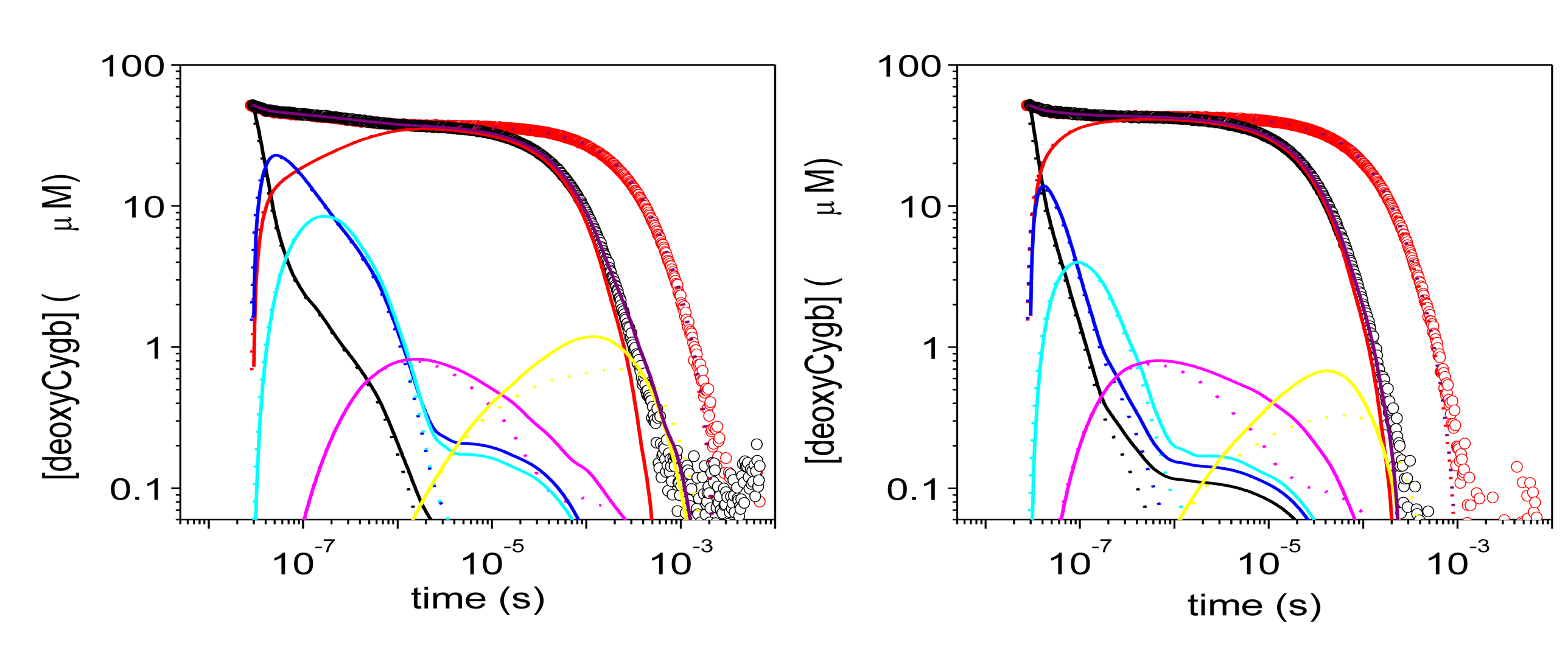

Supplement: Figure S11 — Representative analysis of CO rebinding to HE7Q Cygb* solutions. Global analysis of the CO rebinding kinetics to HE7Q Cygb* solutions at T = 10°C (left) and T = 40°C (right), equilibrated with 1 atm CO (black circles) and 0.1 atm CO (red circles). The fits (purple lines) are superimposed to the experimental data (circles). In the figures we have also reported the time course of the other relevant species in the scheme in Figure 2, at 1 atm CO (solid lines) and 0.1 atm CO (dotted lines): (Cygbp:CO)1 (black), (Cygbp:CO)2 (blue), (Cygbp:CO)3 (cyan), (Cygbp:CO)4 (magenta), (Cygbp:CO)5 (yellow), (Cygbh:CO)6 (dark blue), Cygbh (green), Cygbp (red). Analysis of the CO rebinding kinetics to HE7Q Cygb* solutions (Figure S10) shows partly inhibited migration pattern through internal hydrophobic cavities in comparison to the one observed for Cygb solutions. The source for this can be found in the higher reactivity of the this mutant (see Table S4). The kinetics at 1 and 0.1 atm CO can be perfectly reproduced over the whole investigated temperature range. (TIF) [file pone.0049770.s012.tif]
