# Supplementary material for: CO Rebinding Kinetics and Molecular Dynamics Simulations Highlight Dynamic Regulation of Internal Cavities in Human Cytoglobin
Source: PLoS One. 2013 Jan 4;8(1):e49770. doi: 10.1371/journal.pone.0049770 (PMC3537629; doi:10.1371/journal.pone.0049770)
Supplement: Table S2 — Comparison between microscopic rate constants for wt Cygb solutions, COCygb gels and Cygb+CO gels from the fit of the flash photolysis data, at 20°C. Activation enthalpies and entropies were estimated from the linear Eyring plots for each rate constant k i in the temperature range 10–40°C. (DOCX) [file pone.0049770.s014.docx]

**Table S2**

Comparison between microscopic rate constants for wt Cygb solutions, COCygb gels and Cygb+CO gels from the fit of the flash photolysis data, at 20 °C. Activation enthalpies and entropies were estimated from the linear Eyring plots for each rate constant *k*_i_ in the temperature range 10-40 °C.

|  |  | **solution** |  |  | **COCygb gel** |  |  | **Cygb+CO gel** |  |
| --- | --- | --- | --- | --- | --- | --- | --- | --- | --- |
|  | *k* | *TΔS*^‡^ @20 °C (kcal/mol) | *ΔH*^‡^  (kcal/mol) | *k* | *TΔS*^‡^ @20 °C (kcal/mol) | *ΔH*^‡^  (kcal/mol) | *k* | *TΔS*^‡^ @20 °C (kcal/mol) | *ΔH*^‡^  (kcal/mol) |
| *k*_-1_ (10^6^ s^-1^) | 14.9 | -5.168±0.003 | -0.575±0.005 | 18.6 | -7.43±0.03 | -0.03±0.03 | 16.5 | -8.1±0.3 | -0.6±0.2 |
| *k*_2_ (10^6^ s^-1^) | 42.0 | -3.3±0.2 | 3.7±0.2 | 20.8 | -0.4±0.4 | 6.9±0.6 | 18.4 | -1.11±0.01 | 6.29±0.01 |
| *k*_-2_ (10^6^ M^-1^s^-1^) | 30.4 | 1.3±0.5 | 8.3±0.5 | 38.4 | 3.4±0.4 | 10.4±0.4 | 16.5 | 0.46±0.228 | 7.9±0.7 |
| *k*_c_ (10^6^ s^-1^) | 15.2 | -2.862±0.005 | 4.650±0.005 | 18.3 | -7.0±0.1 | 0.4±0.1 | 22.2 | -1.45±0.03 | 5.85±0.03 |
| *k*_-c_ (10^6^ s^-1^) | 9.5 | -0.18±0.02 | 7.61±0.02 | 15 | -5.1±0.2 | 2.4±0.2 | 5.4 | -6.4±0.4 | 1.7±0.4 |
| *k*_d_ (10^6^ s^-1^) | 8.1 | 1.6±0.1 | 9.4±0.1 | 6.1 | -5.5±0.1 | 2.6±0.1 | 8.1 | -4.9±0.4 | 2.9±0.4 |
| *k*_-d_ (10^6^ s^-1^) | 1.6 | -1.2±0.1 | 7.6±0.1 | 2.3 | -7.2±0.1 | 1.4±0.1 | 0.5 | -6.7±0.1 | 2.8±0.1 |
| *k*_e_ (10^6^ s^-1^) | 1.7 | -2.6±0.2 | 6.2±0.2 | 4.9 | -7.55±0.01 | 0.62±0.01 | 0.87 | -5.88±0.05 | 3.30±0.05 |
| *k*_-e_ (10^6^ s^-1^) | 0.38 | -3.2±0.2 | 6.5±0.2 | 0.4 | -6.3±0.4 | 3.4±0.4 | 0.049 | 1.62±0.04 | 12.47±0.05 |
| *k*_f_ (10^6^ s^-1^) | 0.26 | -1.4±0.2 | 8.5±0.2 | 0.1 | -8.1±0.3 | 2.2±0.3 | 0.007 | -11.18±0.03 | 0.8±0.4 |
| *k*_-f_ (10^2^ s^-1^) | 58.9 | 7.4±0.4 | 19.5±0.4 | 37.6 | -5.9±0.9 | 6.5±0.9 | 16.5 | -7.4±0.9 | 5.4±0.9 |
| *k*_a_ (10^2^ s^-1^) | 3.1 | 6.7±0.3 | 20.5±0.3 | 25.6 | 1.1±0.9 | 14±1 | 5.48 | -5.3±0.5 | 8.1±0.6 |
| *k*_-a_ (10^2^ s^-1^) | 3.35 | 4.3±0.9 | 13.8±0.9 | 103 | 2.6±0.9 | 14.4±0.9 | 1.68 | -9.2±0.9 | 4±1 |
| *k*_b_ (10^2^ s^-1^) | 1.49 | -2.7±0.5 | 11.4±0.5 | 3.3 | -3±1 | 11±1 | 1.71 | -5.51±0.03 | 8.63±0.03 |
| *k*_-b_ (10^2^ s^-1^) | 0.018 | -1±1 | 15.3±0.3 | 3.6 | -6±1 | 8±1 | 0.01 | 5.20±0.01 | 22.31±0.01 |
| *k*_3_ (10^2^ s^-1^) | 12.3 | -2.7±0.7 | 10.3±0.7 | 13 | -3.8±0.4 | 9.1±0.4 | 27.5 | -5.9±0.4 | 6.7±0.4 |
| *k*_5_ (10^2^ s^-1^) | 6.3 | -8.8±0.2 | 4.6±0.2 | 6.9 | -8.7±0.4 | 4.7±0.4 | 0.39 | 4.7±0.6 | 19.7±0.6 |
| *k*_ON_(10^6^M^-1^s^-1^) | 6.3 | - | - | 12.4 | - | - | 5.5 | - | - |
