# Supplementary material for: CO Rebinding Kinetics and Molecular Dynamics Simulations Highlight Dynamic Regulation of Internal Cavities in Human Cytoglobin
Source: PLoS One. 2013 Jan 4;8(1):e49770. doi: 10.1371/journal.pone.0049770 (PMC3537629; doi:10.1371/journal.pone.0049770)
Supplement: Table S3 — Activation free energies at 20°C for Cygb solutions and gels. (DOCX) [file pone.0049770.s015.docx]

**Table S3**

Activation free energies at 20 °C for Cygb solutions and gels.

|  | **solution** | **COCygb gel** | **Cygb+CO gel** |
| --- | --- | --- | --- |
|  | *ΔG*^‡^ @20 °C (kcal/mol) | *ΔG*^‡^ @20 °C (kcal/mol) | *ΔG*^‡^ @20 °C (kcal/mol) |
| *k*_-1_ | 4.59±0.01 | 7.40±0.06 | 7.5±0.2 |
| *k*_2_ | 7.0±0.4 | 7±1 | 7.40±0.01 |
| *k*_-2_ | 7±1 | 7.0±0.8 | 7.4±0.7 |
| *k*_c_ | 7.51±0.01 | 7.4±0.3 | 7.3±0.03 |
| *k*_-c_ | 7.79±0.03 | 7.5±0.4 | 8.1±0.4 |
| *k*_d_ | 7.9±0.2 | 8.1±0.2 | 7.9±0.4 |
| *k*_-d_ | 8.8±0.2 | 8.6±0.2 | 9.5±0.1 |
| *k*_e_ | 8.8±0.4 | 8.17±0.01 | 9.20±0.05 |
| *k*_-e_ | 9.7±0.4 | 9.7±0.8 | 10.85±0.05 |
| *k*_f_ | 9.9±0.4 | 10.3±0.6 | 11.9±0.4 |
| *k*_-f_ | 12.1±0.8 | 12±2 | 13±1 |
| *k*_a_ | 13.8±0.6 | 13±2 | 13.5±0.6 |
| *k*_-a_ | 13.8±0.2 | 12±2 | 14±1 |
| *k*_b_ | 14.1±0.9 | 14±2 | 14.15±0.03 |
| *k*_-b_ | 17±3 | 14±2 | 17.11±0.01 |
| *k*_3_ | 13±1 | 12.9±0.9 | 12.5±0.4 |
| *k*_5_ | 13.4±0.5 | 13.4±0.7 | 15.0±0.6 |
