# Supplementary material for: CO Rebinding Kinetics and Molecular Dynamics Simulations Highlight Dynamic Regulation of Internal Cavities in Human Cytoglobin
Source: PLoS One. 2013 Jan 4;8(1):e49770. doi: 10.1371/journal.pone.0049770 (PMC3537629; doi:10.1371/journal.pone.0049770)
Supplement: Table S4 — Microscopic rate constants for HE7Q Cygb* solutions from the fit of the flash photolysis data, at 20°C. Activation enthalpies and entropies were estimated from the linear Eyring plots for each rate constant k i in the temperature range 10–40°C. (DOCX) [file pone.0049770.s016.docx]

**Table S4**

Microscopic rate constants for HE7Q Cygb* solutions from the fit of the flash photolysis data, at 20 °C. Activation enthalpies and entropies were estimated from the linear Eyring plots for each rate constant *k*_i_ in the temperature range 10-40 °C.

|  |  |  |  |  |
| --- | --- | --- | --- | --- |
|  | *k* | *TΔS*^‡^ @ 20 °C (kcal/mol) | *ΔH*^‡^  (kcal/mol) | *ΔG*^‡^ @ 20 °C (kcal/mol) |
| *k*_-1_ (10^6^ s^-1^) | 12.0 | -7.14±0.03 | 0.51±0.03 | 7.65±0.06 |
| *k*_2_ (10^6^ s^-1^) | 37.7 | -2.88±0.06 | 4.10±0.06 | 6.98±0.12 |
| *k*_-2_ (10^6^ M^-1^s^-1^) | 122.7 | -0.10±0.03 | 6.19±0.03 | 6.30±0.06 |
| *k*_c_ (10^6^ s^-1^) | 66.4 | -6.85±0.01 | -0.19±0.01 | 6.66±0.02 |
| *k*_-c_ (10^6^ s^-1^) | 23.7 | -0.46±0.21 | 6.78±0.21 | 7.25±0.42 |
| *k*_d_ (10^6^ s^-1^) | 8.2 | -5.97±0.11 | 1.91±0.12 | 7.87±0.23 |
| *k*_-d_ (10^6^ s^-1^) | 8.5 | -8.28±0.12 | -0.45±0.12 | 7.84±0.24 |
| *k*_e_ (10^6^ s^-1^) | 0.45 | 0.98±0.43 | 10.59±0.45 | 9.61±0.88 |
| *k*_-e_ (10^6^ s^-1^) | 0.094 | 0.08±0.35 | 10.52±0.36 | 10.44±0.71 |
| *k*_f_ (10^6^ s^-1^) | 0.074 | -10.39±0.08 | 0.23±0.08 | 10.62±0.16 |
| *k*_-f_ (10^2^ s^-1^) | 100.2 | -6.15±0.48 | 5.63±0.49 | 11.78±0.97 |
