# Supplementary material for: CO Rebinding Kinetics and Molecular Dynamics Simulations Highlight Dynamic Regulation of Internal Cavities in Human Cytoglobin
Source: PLoS One. 2013 Jan 4;8(1):e49770. doi: 10.1371/journal.pone.0049770 (PMC3537629; doi:10.1371/journal.pone.0049770)
Supplement: Table S5 — Data collection and refinement statistics for HE7Q Cygb*. (DOCX) [file pone.0049770.s017.docx]

**Table S5**. Data collection and refinement statistics for HE7Q Cygb*

| **Data collection** |  |
| --- | --- |
| Space group | *P*2_1_2_1_2_1_ |
| Cell dimensions | *a* = 48.8 (Å),  *b =* 70.1 (Å), *c* = 102.1 (Å) |
| Resolution limits | 57.7-2.8 (Å) |
| Observations | 31,575 |
| Unique reflections | 9,092 |
| Completeness (%) overall | 99.8 % (100.0 %)^a^ |
| R-merge*^b^* (%) | 10.7 (42.0) |
| I/σ(I) (outer shell) | 10.9 (3.3) |
| Multiplicity | 3.5 (3.6) |
| **Refinement** |  |
| R-factor*^c^*/R-free*^d^* | 20.7 % / 27.7 % |
| N° of protein residues / mean B-factor:  Subunit A  Subunit B  N° of heme groups / mean B-factor:  N° of cyanide molecules / mean B-factor:  N° of ferricyanide molecules / mean B-factor:  N° of acetate molecules / mean B-factor:  N° of water molecules / mean B-factor: | 154 (18-171) / 22.5 (Å^2^)  154 (18-171) / 21.1 (Å^2^)  2 / 18.8 (Å^2^)  2 / 39.5 (Å^2^)  2 / 37.1 (Å^2^)  1 / 51.4 (Å^2^)  22 /11.8 (Å^2^) |
| R.m.s. deviation from ideality:  bond lengths  bond angles | 0.007 (Å)  1.02° |
| Ramachandran plot:  residues in most favored regions  residues in additional allowed regions | 93.5 %  6.5 % |

*^a^* Values in parentheses are for highest-resolution shell (2.95-2.80 Å).

*^b^* R-merge =Σ_h_Σ_i_ | I_hi_ – <I_h_> | / Σ_h_Σ_i_ I_hi_.

*^c^* R-factor = Σ_h_ ||F_obs_| - |F_calc_|| / Σ |F_obs_| where F_obs_ and F_calc_ are the observed and calculated structure factor amplitudes, respectively.

*^d^* R-free is calculated on 10% of the diffraction data, which were not used during the refinement.
