# Supplementary material for: CO Rebinding Kinetics and Molecular Dynamics Simulations Highlight Dynamic Regulation of Internal Cavities in Human Cytoglobin
Source: PLoS One. 2013 Jan 4;8(1):e49770. doi: 10.1371/journal.pone.0049770 (PMC3537629; doi:10.1371/journal.pone.0049770)
Supplement: Table S6 — Contribution (%) of the first 10 eigenvectors derived from essential dynamics to the conformational flexibility of the protein backbone. (DOCX) [file pone.0049770.s018.docx]

**Table S6**. Contribution (%) of the first 10 eigenvectors derived from essential dynamics to the conformational flexibility of the protein backbone.

| Eigenvector | *Cygb_h_* | *Cygb_p_* (HE7Q) | *Cygp_h_*(3AG0) | O_2_Cygb(HE7Q) | O_2_Cygb(3AG0) |
| --- | --- | --- | --- | --- | --- |
| 1 | 22.3 | 25.3 | 24.4 | 32.3 | 19.2 |
| 2 | 6.7 | 17.9 | 9.7 | 11.6 | 11.9 |
| 3 | 6.1 | 5.8 | 8.3 | 5.7 | 8.1 |
| 4 | 5.1 | 4.6 | 7.0 | 3.8 | 6.3 |
| 5 | 3.8 | 4.4 | 4.7 | 3.6 | 4.4 |
| 6 | 3.5 | 3.6 | 3.3 | 3.1 | 3.2 |
| 7 | 3.1 | 2.8 | 2.9 | 2.4 | 2.8 |
| 8 | 2.7 | 2.2 | 2.6 | 2.0 | 2.2 |
| 9 | 2.6 | 1.8 | 2.2 | 1.6 | 2.1 |
| 10 | 2.3 | 1.8 | 1.8 | 1.5 | 1.8 |
| Cumulative | 58.2 | 70.2 | 66.9 | 67.6 | 62.0 |
